# Ambiguity in the processing of Mandarin Chinese relative clauses: One factor cannot explain it all

**DOI:** 10.1371/journal.pone.0178369

**Published:** 2017-06-08

**Authors:** Michael P. Mansbridge, Katsuo Tamaoka, Kexin Xiong, Rinus G. Verdonschot

**Affiliations:** 1 Department of Japanese Language and Culture, Nagoya University, Nagoya, Aichi, Japan; 2 Waseda Institute for Advanced Study (WIAS), Waseda University, Tokyo, Japan; University of Leicester, UNITED KINGDOM

## Abstract

This study addresses the question of whether native Mandarin Chinese speakers process and comprehend subject-extracted relative clauses (SRC) more readily than object-extracted relative clauses (ORC) in Mandarin Chinese. Presently, this has been a hotly debated issue, with various studies producing contrasting results. Using two eye-tracking experiments with ambiguous and unambiguous RCs, this study shows that both ORCs and SRCs have different processing requirements depending on the locus and time course during reading. The results reveal that ORC reading was possibly facilitated by linear/temporal integration and canonicity. On the other hand, similarity-based interference made ORCs more difficult, and expectation-based processing was more prominent for unambiguous ORCs. Overall, RC processing in Mandarin should not be broken down to a single ORC (dis)advantage, but understood as multiple interdependent factors influencing whether ORCs are either more difficult or easier to parse depending on the task and context at hand.

## Introduction

When comparing sentence processing strategies between languages, several cross-linguistic differences have been observed, making it unclear whether strategies differ across languages. Occasionally, competing models make dichotomous predictions for a certain language, for example, when processing relative clauses in Mandarin Chinese (henceforth “Mandarin”). In Mandarin, past studies are divided on their support for different contending models. In the present study, we employ eye-tracking to empirically investigate several relative clause processing models within different contexts to explore their interrelationships. First, we briefly introduce the topic of relative clauses, followed by several available processing accounts, and then discuss how these models might function in Mandarin.

### Relative clauses

Example of a Mandarin Chinese relative clause: SRC / ORC

[*t*_i_ *Zhǐzé Shìzhǎng / Shìzhǎng Zhǐzé t*_i_      *De*] *Jìzhě*_i_      *Cǎifǎng-Le      LiNà*

[*t*_*i*_ criticize mayor / mayor criticize    *t*_*i*_ REL] reporter_*i*_ interview-ASP Li Na

‘The reporter_i_ [who *t*_i_ criticized the mayor/the mayor criticized *t*_i_] interviewed Li Na.’

In this paper only subject-extracted relative clauses (SRC) and object-extracted relative clauses (ORC) are discussed. Relative clauses (RC) are often discussed in terms of *wh*-movement and filler-gap dependencies [1,2], where the *filler* is the co-indexed element that fills the *gap* created at the trace (i.e., *t*_i_) of the *wh*-movement. For prenominal (i.e., head-final) Mandarin, not only are these general principles of filler-gap dependencies assumed to be consistent [3], Zhang [4] also provides a framework detailing how gapped RCs are syntactically distinct from non-gapped pseudo-relative clauses. Furthermore, Su, Lee, and Chung [5] showed that a subset of Mandarin speaking aphasic patients exhibited agrammatic comprehension for SRCs but not for ORCs. Along with previous findings, they argued that this effect is best explained by the *Trace-Deletion Hypothesis* [6]. That is, without a gap for the head noun to fill, aphasics cannot comprehend SRCs, but are able to process ORCs due to their canonical agent-to-theme ordering. Considering these points, we opted to avoid any non-gapped interpretation for relative clauses in Mandarin.

In Mandarin, word order differences in relation to the RC and head noun ordering (e.g., post-nominal and prenominal) are relatively unique; ORCs are canonically SVO while SRCs are non-canonical VOS. Typically in languages which have a canonical word order distinction between RC conditions, SRCs are found to be canonical. This makes Mandarin RCs relatively rare among languages, since they display an infrequent, reversed pattern [7,8]. Additionally, Mandarin does not have a relative pronoun; instead, it typically marks the clause in a general manner with the relativizer “*De*” [3,9] which can appear in numerous other constructions besides an RC (see [10]). Since Mandarin is a prenominal RC language and does not mark the RC at the left boundary, temporary ambiguity of clause type exists during the initial reading of the RC. This means that a matrix clause interpretation is often initially incorrectly taken instead of a correct RC interpretation. This ambiguity, however, can be resolved partially at the relativizer, where it becomes clearer that the current clause is likely not a matrix clause but instead is more likely an RC or another clause bearing a similar structure. It is worth noting that besides the RC interpretation, other clause types may also be available at the relativizer, such as appositives, fact-clauses or even pseudo-relative clauses. Accordingly, interpreting a clause as an RC may be best at the head noun for externally-headed RC constructions in Mandarin. This issue of clause-type ambiguity has been a major issue in the literature dealing with RC processing in East Asian languages.

### Relative clause processing

The focus of RCs in psycholinguistic studies has been primarily built upon the processing asymmetry between SRCs and ORCs, specifically, that SRCs are seemingly easier to process and comprehend. According to Hawkins [11], one explanation for why ORCs are more difficult is that they are inherently more structurally complex compared to SRCs. This difference has also been explained by language universal theories such as the *Noun Phrase Accessibility Hierarchy* (NPAH) [12,13] which states that not only should SRCs be acquired first prior to ORCs, the subject gap is in a more accessible syntactic position, thus making it easier to process and comprehend SRCs compared to ORCs. NPAH, however, was not supported for the L1 acquisition of Mandarin RCs by children [14]. The difficulty with ORCs has been documented across a range of varying languages using different experimental methodologies, and taken together this provides strong evidence for a cross-linguistic SRC advantage. Though these studies support the claim that ORCs are generally more difficult than SRCs, explanations for this phenomenon are not yet formed under a single processing account. This is especially true in Mandarin. Currently, there is a heated debate surrounding the processing preference for RCs in Mandarin which started with the findings of Hsiao and Gibson [15] who argued for an ORC advantage due to storage-based resources (i.e., memory load) within the RC structure (see [16]). Since then, there have been numerous attempts to either refute these claims or provide support for the ORC advantage. Previous studies supporting an SRC advantage have used eye-tracking [17] and self-paced reading [18,19,20], and studies supporting an ORC advantage have used eye-tracking [21,22], event-related potentials [23,24], the maze task [25], and self-paced reading [26,27,28]. The amount of support for each claim using a variety of methods makes this issue highly contested. In the following sections, we describe several existing RC processing models as well as their diverging results or predictions on Mandarin RCs.

#### Expectation-based processing

Models involving computations on either or both language frequency and collocations can be divided into several different accounts. Briefly summarizing, *suprisal* [29,30] involves confirming or refuting the parser’s expectations at each word or phrase, *entropy reduction* [31] involves the uncertainty in the remainder of the sentence, and *canonicity* [32] involves the influence of both structural frequency and regularity from the canonical order of a language. Some researchers, however, have suggested to not adopt approaches looking at either too fine-tuned or coarse-grain approaches dealing with language exposure [33].

Under the general framework of expectation-based processing, sentence processing is guided and facilitated by the input of a language (i.e., frequency), and briefly stated, these models predict that: (1) The more common structure should be easier to initially construct, and (2) expectations on upcoming structures are based on language exposure. When these expectations are not met, processing difficulty arises [29,30]. Since ORCs are typically observed to be less frequent in corpora than SRCs (see [34] for statistical frequencies and *suprisal* predictions and [35] for *entropy reduction* predictions for Mandarin), these models predict that ORCs should be more difficult to process, given that their structure is less expected. For temporarily ambiguous RCs, these predictions on the RC structural frequency cannot be established until the parser becomes aware of the RC structure. Considering that Mandarin RCs are ambiguous and that initial word order for ORCs is canonical, expectation models can actually facilitate the initial reading of an ORC. This facilitation would occur up to the relativizer, where the expectation of a canonical ordered matrix clause would prospectively be disconfirmed. Here, ORCs would be more difficult than SRCs resulting from greater surprisal [29,30]. Qiao, Shin, and Forster [25] provided evidence of this account using the maze task. They found an initial ORC advantage within the RC which was later reversed at the relativizer position. Vasishth, Chen, Li, and Guo [20], using self-paced reading, also revealed effects of an ORC disadvantage starting at the relativizer position which offers additional support for surprisal effects. Conversely, Packard, Ye, and Zhou [23] and Sun, Bever, Cheng, Schmidt, and Seifert [24] found greater P600 and N400 event related potentials (ERP) at the relativizer for ambiguous, subject-modified SRCs alluding to greater processing difficulty for SRCs in conflict with expectation-based processing. However, these results do not completely negate other studies since parsing expectations may not always be observable.

On the other hand, if the RC is unambiguous (i.e., the parser correctly assumes an RC interpretation) then SRCs should be easier to parse. This has been corroborated by Jäger, Chen, Li, Lin, and Vasishth [17] who demonstrated, using both self-paced reading and eye-tracking under an unambiguous context (i.e., syntactic cues were inserted to reduce the level of ambiguity), that RTs within the relative clause, excluding the relativizer, were significantly longer for ORCs. This compelling result demonstrates that if the reader is aware of an upcoming relative clause, SRCs will be easier to process in Mandarin. In contrast with the above finding, Gibson and Wu [27], Lin [28], and Vasishth et al. [20] observed an opposing ORC advantage when using discourse to prime participants for an upcoming RC structure; however, both Lin [28] and Vasishth et al. [20] argued that this could be primarily attributed to canonical thematic priming.

#### Memory-based constraints

During sentence comprehension, the parser is constantly assigning case and thematic values to nouns as well as integrating each new syntactic dependency into the structure and reactivating linked words with their antecedents. At the gap position within the RC, the mental parser performs a search for the head noun dependency (i.e., the filler) to retrieve and integrate it with the gap. The difficulty surrounding integrating the filler with its co-indexed dependent is thought to result from the decay of a dependent’s activation in memory [16,36]. While it is generally agreed that activation will decay as more discourse referents are introduced in structure, it is still unclear in which exact manner integration occurs.

One prominent model for integration is Gibson’s [16] *Dependency Locality Theory* (DLT). Within this model, Gibson describes each syntactic dependency as carrying a unit in working memory. This has an effect during the comprehension of a sentence because the parser is (i) constantly predicting the upcoming syntactic dependencies to complete a grammatical sentence (i.e., storage-based resources) and (ii) memory units also apply to the number of intervening referents between filler-gap dependencies. The particularities of DLT suggest that integration performs a strictly linear search in memory for a co-indexed referent and that integration can be assumed to be more difficult as the distance increases. In English, DLT predicts a greater processing demand for ORCs based on the number of intervening dependencies between the filler and gap, when positing the gap at the RC verb. However, this prediction is reversed for prenominal languages like Mandarin since the distance between filler-gap dependencies is greater for SRCs. For the storage-based component of DLT, an ORC advantage can initially be predicted in Mandarin if the clause is misconstrued as a canonical matrix clause, thus initially predicting fewer syntactic heads [15,25].

**Fig 1:**
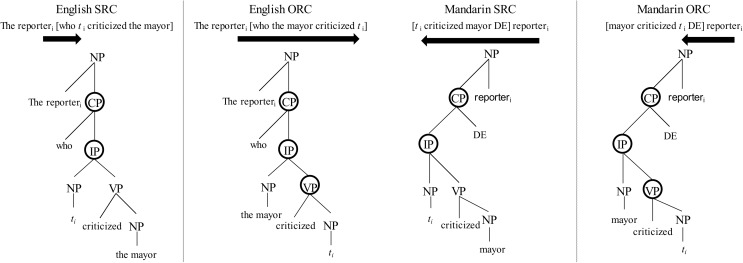
Basic syntactic structure of SRCs and ORCs in English and Mandarin. The linear/temporal integration metric is described by the black horizontal arrow (longer arrows indicate increased cost). The structural-phrase metric is described by the circles in in the syntactic structure (more circles indicate increased cost).

In a similar vein, Lewis and Vasishth’s [36] *activation-based model*, based within the scope of the *Adaptive Control of Thought–Rational* (ACT-R) model [37], proposes that the decay of the initial activation increases as a function of time (i.e., temporal locality). Vasishth and Lewis [38] also contend that successive activations on the current input has the potentiality of creating *antilocality* effects such that a condition with a higher activation level will lead to anticipatory facilitation in reading speed. Concerning integration, the activation-based model makes similar predictions as DLT. Yet, these models differ from the *Structural-Hierarchy* model [39], which defines decay by the number of intervening syntactic phrases within syntactic structure hierarchy. Since SRCs have fewer syntactic heads intervening between the filler and gap, ORCs would be more difficult during integration. See Fig 1 for an illustration of these models.

In support of DLT, Packard et al. [23] found increased P600 ERP responses for the SRC condition at the relativizer and head noun in Mandarin and attributed it to a greater processing demand for SRCs during integration [40,41]. They claimed that the relativizer has the potential to satisfy the categorical selectional restriction of the RC verb; as such, the relativizer can serve as a substitute for the filler during integration. This notion is also built upon evidence from Mandarin, as well as Korean and Japanese. These are languages which allow for null-head RC structures [42,43] which could necessitate that the relativizer needs to generate a head NP and take upon the responsibilities of integration without carrying specific lexical information that the missing head would carry. As such, they suggested that even in headed RCs, the relativizer can still act in this manner. Similar to Packard et al. [23], Sun et al. [24] found an increased N400 at the relativizer and head noun for SRCs, which may suggest that the metrics of integration for Mandarin are indeed based on linear/temporal locality rather than locality in syntactic structure [39]. Sun et al. [24], however, instead argued that the surface canonical word order creates a garden path effect which only initially benefits ORCs. When using eye-tracking, it was shown that Sung, Cha, Tu, Wu, and Lin [21] and Jäger et al. [17] yielded conflicting results. Specifically, an ORC advantage was found in ambiguous RCs [21] while an ORC disadvantage was found in unambiguous RCs [17]. Considering the number of conflicting findings, more studies are needed to determine whether ORCs are easier to process due to integration or are only easier due to a garden path effect resulting from their similarity to the canonical order of Mandarin within ambiguous RCs.

Lewis and Vasishth [36,38] note a third interactive feature in their memory constraint model based upon the interference of similar referents being held in memory, i.e., *similarity-based interference* within the framework of *cue-based retrieval*. In relation to ACT-R [37], it is described that the activation level of a given item is also influenced by the number of other items sharing overlapping features (e.g., animacy, syntactic position, gender, number) surrounding it within a sentence. As the number of similar items increase, the activation level for each of these items will decrease causing a *fan effect*. Upon encountering an item (e.g., a pronoun, reflexive, or verb) which necessitates a dependent with a specific set of cue features (e.g., +animate, +female, +singular, +subject), a *retrieval-cue* process will be initiated to select the grammatically correct antecedent matching the cue features in memory, i.e., the target item. This process will be more difficult if there are distractor items matching the cues but are, nevertheless, ungrammatical antecedents. A distractor item can either proactively or retroactively reduce the activation level of the target depending on whether if it precedes or follows the target. While Lewis and Vasishth’s model does not make a strong claim for similarity interference at the matrix verb for RC processing (they instead argued for stronger effects of similarity interference at the embedded RC verb in English [36]), we consider that there is a possibility for it to occur in Mandarin despite the lack of subject-verb agreement features. Similarity interference, however, has mixed findings, sometimes showing inhibition or facilitation depending on the context (see [44] for a comprehensive meta-analysis overview).

In a recent study by Patil, Vasishth, and Lewis [45], similarity interference was argued for reflexive anaphora in English despite previous notions of reflexive anaphoric binding in English arguing for a purely structural-based account (i.e., no violation to Binding Principal A). They [45] argued that the lack of inhibitory interference effects in previous studies can be attributed to those studies using object-role distractors instead of subject-role when the antecedent required a +subject feature. Therefore, if the distractor shares the +subject feature, processing inhibition for similarity-based interference can be observed during reflexive anaphora. In terms of the processing differences between SRCs and ORCs, Gordon and colleagues [46,47] claimed that ORC difficulty at both the embedded and matrix clause verb may be explained by similarity interference. When using eye-tracking [47] it was observed that ORC difficulty appeared within the RC and at the matrix verb. While they [47] had tested for both RC condition and the effect of noun type (i.e., proper noun and general nouns), processing differences based on both were found only within the RC, and the matrix verb was only observed to have ORC difficulty. Thus, when a predicate necessitates a subject lacking agreement features beyond animacy, the subject within the ORC may still potentially provide an interference account at the matrix verb. Considering the importance matching grammatical features for similarity-interference in other studies [48], we argue that similarity-based interference should be extended to matrix predicates for Mandarin RC processing under this premise. Accordingly, since subject-modified ORCs in Mandarin have two grammatical subjects (i.e., RC and matrix clause subjects) prior to the matrix clause verb, we suggest that the RC subject should proactively cause a fan effect for the matrix clause subject. Therefore, when the matrix verb retrieves its subject using the retrieval-cues +subject and +animate, ORC sentences should be more difficult in comparison to SRC sentences since the SRC noun instead has the feature +object. Consequently, we suggest that similarity-based interference can predict ORC processing difficulty in Mandarin. For more detail on similarity interference see [49].

### Current study

Regarding RC processing in Mandarin, recent studies have revealed that the ORC advantage is primarily seen in ambiguous contexts while unambiguous contexts favour SRC processing. Yet, these studies have not fully addressed ambiguity as an experimental factor. Accordingly, the present study will further investigate these results to provide a more detailed account of processing within ambiguous and unambiguous RCs. Our first experiment sets out to replicate previous findings within an ambiguous design using two different experimental tasks. In Experiment 2, we modify Jäger et al.’s [17] items to either include the determiner + classifier phrase (i.e., attenuated ambiguity) or exclude it (i.e., ambiguous) to determine if ORCs are only facilitated in ambiguous contexts. As such, we explore the relevancy of canonicity, linear/temporal integration metrics, expectation-based processing and similarity interference for RC processing in Mandarin Chinese.

## Experiment 1

For Experiment 1, using a strictly ambiguous RC structure, we sought to replicate recent eye-tracking findings such that ORCs would be easier to process than SRCs within the RC. We also sought to demonstrate that ORCs become more difficult to parse after the reading of the head noun as specified by similarity-based interference.

In Experiment 1 we employed both a plausibility judgment task (i.e., a sentential judgment task on the overall plausibility of the event denoted by the sentence, not its grammaticality) and a traditional verification judgment task (i.e., post-sentence comprehension/verification questions) on Mandarin RCs using a slightly irregular Mandarin RC type containing two proper names as the RC noun and head noun. The plausibility task was added to determine if any result obtained was influenced by task artefacts. According to Caplan, Chen, and Waters [50], the use of comprehension or verification questions may be more cognitively demanding than what is required to process and understand the sentence. They attribute this to participants attempting to rehearse the sentence while reading it for the purpose of answering the post-sentence question. In contrast, their plausibility task generated less BOLD signal response using fMRI than their verification task while still having activation in regions responsible for syntactic processing similar to their verification task. Considering that the majority of the studies investigating Mandarin RC processing have used traditional comprehension questions, we investigate the effect of task on RC processing as a secondary, minor objective of Experiment 1. In other words, we would like to determine how the ORC (dis)advantage is influenced by the task participants must attend to during the reading of Mandarin RCs. At the very least, we expect to find increased reading times in the verification task in comparison to the plausibility task in support of Caplan et al. [50].

### Materials and methods

#### Participants

Thirty-two native speakers of Mandarin Chinese, all originating from Mainland China, were recruited from Nagoya University, Japan, but five were removed due to extensive calibration difficulties (N = 27; Female = 17). The mean age of the participants was 24.5 years (range 22–30.5 years).

#### Materials

Thirty-two experimental items were created. Each item contained an ambiguous relative clause that only modified the matrix subject. Each RC had two variants: an ORC and its SRC counterpart. Items were counterbalanced to ensure that participants would only see one condition of each item per task session. Half of the participants first undertook the plausibility task before the verification task and vice versa.

All experimental items were plausible. The length of each noun, verb, and adverb was two simplified Chinese characters. All nouns were set as an animate proper name; for example “*Lǐ Fāng*” and “*Liáng Yuán*”. The majority of these names were taken off of a list of common Chinese names from the National Citizen Identity Information Center [全国公民身份号码查询服务中心]. Furthermore, the gender of the nouns was controlled such that male and female names were distributed equally. Animacy has been a well-known issue for RC processing in Mandarin with ORCs having the preference of having an inanimate head and animate RC noun whereas SRCs are preferred to have an inanimate RC noun and animate head. Within animate-animate contexts, however, subject-modified SRCs appeared to be more frequent in comparison to subject-modified ORCs [51,52]. Therefore, it is possible that by using only animate nouns this may create a slight ORCs disadvantage. However, RCs with two proper nouns should be relatively rare for both RC types, so we believe animacy effects should not be problematic for ORCs. While the frequency of the verbs was also controlled, stroke count was not controlled for the nouns, adverbs and verbs.

As seen below, each word, besides the particle “*Le*”, has been coded: N1 stands for the RC noun, V1 is the RC verb, DE is the relativizer, N2 is the head noun, ADV is an adverb, and V2 identifies the matrix clause verb with the aspect marker “*Le*”. In the plausibility task, an equal number of implausible RC distractors were also shown, see below for an example. In the verification task, half of the questions were true or correct probes and the other half were false or incorrect probes. See the Appendix for a list of all experimental stimuli.

SRC / ORC items of Experiment 1

        V1        N1      /  N1        V1        DE        N2        ADV        V2

[*t*_i_  *Yāoqǐng LǐFāng* / *LǐFāng Yāoqǐng t*_i_    *De*] *LiángYuán*_i_ *Gāngcái      Chídào Le*

[*t*_i_  invited Li Fang / Li Fang invited *t*_i_ REL] Liang Yuan  just.now      late ASP

‘Liang Yuan [who invited Li Fang / Li Fang invited] was late just now.’

Example of Implausible RC Distractor

[*Zhāng Wěi Chī Le De*] *Li Qiáng Yǐjīng Huí Jiā Le*

[Zhang Wei ate REL] Li Qiang already returned

‘Li Qiang who Zhang Wei ate already went home.’

#### Procedure

Experiment 1 involved exposing participants to two different tasks. Each task was done in a separate session and half of the participants first took one task before the other. In both tasks, items were counterbalanced such that no participant would see the same item twice within a single task, nor would they see an identical item between tasks. Stimulus sentences were displayed horizontally on the centre left of a 17-inch Mitsubishi LCD monitor at a distance of 70 cm from the head and chin rest mount. All characters were displayed in Chinese MingLiU 30pt. At this distance, each character subtended a visual angle of 2.5°. Eye-movements were recorded using an EyeLink 1000 Core System. Prior to the experiment, participants were instructed in Mandarin that they would be reading Mandarin sentences displayed one at a time on a computer monitor, and were given the opportunity to ask questions about the procedure. Prior to each session, the camera was calibrated by a 9-point calibration method and subsequent validation. Calibration was periodically repeated throughout each session after block sessions (eight items).

For the plausibility task, participants were instructed in Mandarin to read each sentence naturally and judge if the sentence meaning was plausible, that is, if the actions or ideas depicted would be able to exist in a real world, everyday setting. If the sentence meaning was plausible, they were instructed to press a button on a gamepad labelled “True”; conversely, if the sentence meaning was not plausible, they were instructed to press a button labelled “False”. Participants were instructed to read and judge each sentence within eight seconds. After pressing either button, the stimulus was immediately removed from the screen. Reading times were measured from the onset of the stimulus to the button press event. Eight practice trials were given to ensure participants understood the task.

For the verification task, only minor changes were made to the procedure. Participants were instructed to read each sentence naturally and that after reading the sentence a comprehension question would appear. Again, participants were asked to read each sentence within eight seconds. When they were finished reading the sentence, participants were instructed to press a button that would replace the sentence with a comprehension/verification question (e.g., did *Li* invite *Liang*?). Reading times were measured from the onset of the stimuli to this button press event. For the question, participants had up to eight seconds to answer. When answering, participants were instructed to press the “True” button for correct or true probes or the “False” button for incorrect or false probes. Eight practice trials were given to ensure participants understood the task. For both tasks, since reading times were measured from the onset of stimuli until the button response events (i.e., judging the plausibility of the sentence or proceeding onto the question) reading times and eye-movements are comparable between the two tasks.

### Results

**Fig 2:**
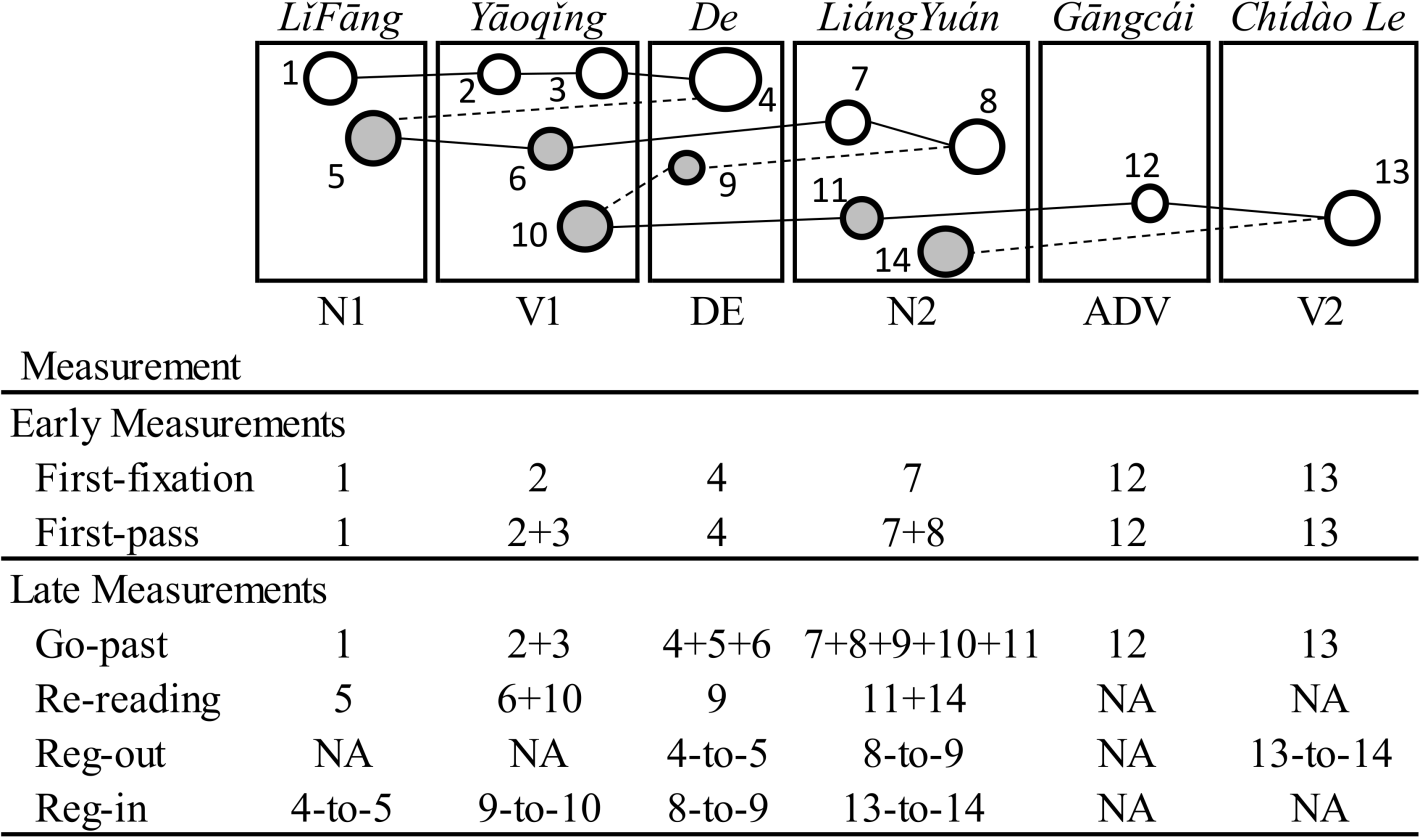
Example of eye-tracking measurements. In this figure, an illustration of eye-fixations and saccadic movements are given for an ORC item from Experiment 1. However, this does not represent actual data collected from Experiment 1. White circles (e.g., 2 and 3) represent fixations that were made during the first-pass through a given region and grey coloured circles (e.g., 6 and 10) represent any fixation that was made after the first-pass (i.e., re-reading) in a given region. Solid lines (e.g., the line between fixations 7 and 8) represent left-to-right saccades (i.e., eye-movements) and dashed lines (e.g., the dashed lines between fixations points 8 and 9) represent backwards right-to-left regressive saccades.

The earliest reading time measure reported here is *first-pass* time, all fixations made within a region from when it is first entered until it is exited. The late measures reported are *re-reading* time, the sum of all fixations in a region after first-pass (total time minus first-pass), and *go-past* time, the combined RT for an interest region (e.g., DE) before it is exited to the right (e.g., N2) for the first time including any regressive readings out of the region to the left (e.g., N1, V1). Go-past times are thus greater than or equal to first-pass times for a region. Regression-in and regression–out (i.e., first-pass regression-out) proportion measures, the total reading time of the sentence and accuracy are also reported. While accuracy for the plausibility task denoted whether the participant accurately judged the experimental RCs as plausible, accuracy for the verification task indicated whether the participant accurately judged the probe to be true/correct or false/incorrect. The interest regions for analyses were the sentence, the RC structure (N1, V1), the relativizer (DE), the head noun (N2), the adverb (ADV), and the matrix verb (V2). Prior to the analyses, eye-fixations were first treated. Fixations below 80 ms were merged into a neighbouring fixation, and the remaining fixations under 80 ms and those exceeding 1000 ms were removed (523 fixations or 2.91%). Refer to Fig 2 for an illustration of these measures (see also [53]).

**Fig 3:**
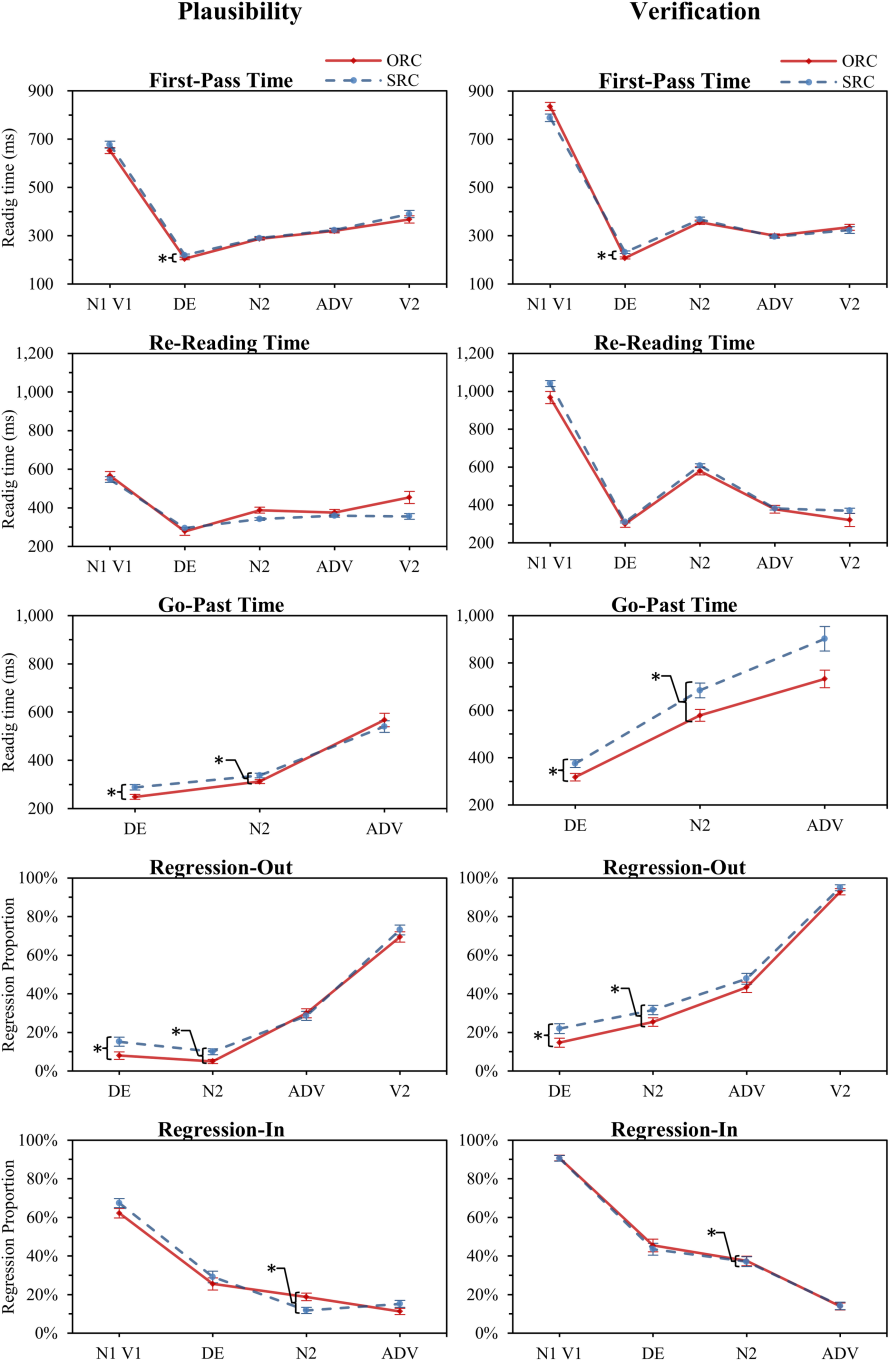
Experiment 1: The trimmed reading times and regression proportions.

A series of linear mixed effect (LME) model analyses [54] were conducted using the *lme4* package [55,56] within R [57]; the RC condition (ORC = -0.5 & SRC = 0.5) and Task type (Plausibility = -0.5 & Verification = 0.5) comprised the fixed effects, and random effects were the subjects and items (see S1 Data). If the interaction of condition:type was significant, a pairwise analysis was conducted. RTs were transformed using natural logarithms for improved normality of the residuals. LME models (a cross-section of random subjects and items with full variance-covariance random effect matrices to those with only varying intercepts [58]) were compared to determine the best fit model using the maximum likelihood technique. This revealed that the simplest model (i.e., random intercepts for both subjects and items) did not differ significantly from (i.e., did not show a lesser fit between) more complex models (i.e., inclusion of random slopes) for all the analyses. Accordingly, we opted to use the simpler model. Analyses of RTs and regression data only included items with correct responses. RT measures with zero RT or regions which were skipped were treated as missing values and were not included in the RT analyses. The *lmerTest* package [59] in R was used to provide RT models with *p*-values using Satterthwaite's approximation for the degrees of freedom. For accuracy and regression proportions, *glmer* (binomial family) within lme4 was used to calculate the *z* distribution using Laplace approximations. Data outliers (RTs only) were trimmed upon ± 2.5 standard deviations of each model (1.65%). Refer to Tables A and B for means and standard errors, and Table C for LME results in S1 Tables. The trimmed reading times, regression proportions are shown for the RC conditions per task in Fig 3 (only the significance for the fixed effect of RC condition is shown).

#### Sentence

*Accuracy*. While both RC condition (*p* = .131) and task type (*p* = .09) were not significant, their interaction (*p* < .01) was significant. This interaction revealed that while within the plausibility task, accuracy between ORCs and SRCs was not significantly different (*p* = .295), ORCs in the verification task had a significantly higher accuracy compared to SRCs (*p* < .001).

*Total reading time*. For the total reading time of the sentence, only task type (*p* < .001) was significant, revealing that the verification task had significantly longer overall reading times compared with the sentences found in the plausibility task. Both RC condition (*p* = .069) and the interaction of RC condition and task type (*p* = .219) did not reveal any significant differences.

#### RC (N1, V1 / V1, N1)

*First-pass RT*. For the first reading of the RC phrase, RC condition (*p* = .388) was not significant. In contrast, task type (*p* < .001) was significant, showing longer reading times for the verification task sentences. Interaction (*p* < .01) of RC condition and task type was significant. This demonstrated that in addition to verification types being significantly longer than the plausibility type counterparts, ORC:Verification (*p* < .05) had significantly longer reading times than SRC:Verification. While the reading times for ORC:Plausibility was numerically less than SRC:Plausibility, this difference was shown to be not significant (*p* = .198).

*Re-reading Time*. For the re-reading of the RC phrase, only task type (*p* < .001) was significant, again showing longer RTs for the verification task items in comparison to the plausibility task. Both RC condition (*p* = .700) and interaction were not significant (*p* = .051) despite RTs for ORC:Verification being numerically less than SRC:Verification.

*Regression-in*. Similar to re-reading time, while RC condition (*p* = .376) and interaction were not significant (*p* = .376), task type (*p* < .001) was significant which revealed a higher probability for the verification task to regress back into the RC compared with the plausibility task.

#### Relativizer (DE)

*First-pass RT*. For the first reading of the relativizer, it was found that RC condition (*p* < .001) revealed a significant difference between RCs with the SRC condition having longer RTs in comparison with the ORC condition. Neither task type (*p* = .165) nor interaction (*p* = .336) were significant.

*Re-reading Time*. No significant effects were found for RC condition (*p* = .541), task type (*p* = .388) and interaction (*p* = .821) during re-reading time.

*Go-past Time*. Prior to moving on to the head noun, go-past RT for RC condition was significant (*p* < .001); it was found that SRCs required longer RTs before moving on. Task type (*p* < .001) was also significant which revealed that the items within the verification task had longer go-past RTs than items in the plausibility task. Interaction (*p* = .386) was not significant.

*Regression-out*. Similar to go-past, both the RC condition (*p* < .01) and task type (*p* < .001) were significant revealing a comparable pattern. SRCs had a higher probability to regress out, and sentences within the verification task also had a higher chance of regressing out of the relativizer. Again, interaction (*p* = .663) of RC condition and task type was not significant.

*Regression-in*. The probability of regressing back into the relativizer was only significant for task type (*p* < .001); it was revealed that there was a higher chance of moving back into the relativizer for items within the verification task. Neither RC condition (*p* = .691) nor interaction were significant (*p* = .295).

#### Head noun (N2)

*First-pass RT*. Upon first entering the head noun, the only significant RT difference was observed for task type (*p* < .001) which revealed significantly longer RTs for the items of the verification task. Neither RC condition (*p* = .608) nor interaction (*p* = .678) had significant results.

*Re-reading Time*. Similar to first-pass, the re-reading of the head only revealed longer RTs for the verification task in comparison with the plausibility task (task type: *p* < .001). Neither RC condition (*p* = .563) nor interaction had significant results (*p* = .066).

*Go-past Time*. Go-past time, however, did reveal a significant difference for RC condition (*p* < .01) which resulted in SRCs having longer go-past times compared to ORCs. Again, task type (*p* < .001) was significant, showing the same pattern of the items of the verification task having longer RTs than those of plausibility. Interaction was not significant (*p* = .099).

*Regression-out*. Similar with go-past RT, the RC condition (*p* < .001) revealed that SRCs had a significantly higher chance of regressing out, and task condition (*p* < .001) showed that the verification task items also had a significantly higher chance. Interaction of the two was not significant (*p* = .205)

*Regression-in*. In contrast to the above, the significant difference between RC conditions (*p* < .05) revealed an opposite pattern. That is, ORCs were more likely to have a regression back into the head noun. However, task type (*p* < .001) demonstrated once more that verification items were more likely to have regressions back into the region than the plausibility items. The interaction of RC condition and task type was significant (*p* < .05); this result demonstrated that it was only within the plausibility task (*p* < .001) that ORCs had a higher probability of having a regression in, while ORCs in the verification task (*p* = .880) did not have a significant difference with their SRC counterparts.

#### Adverb (ADV)

*First-pass RT*. At the adverb, only task type (*p* < .001) was significant. However, at this position, plausibility items had longer RTs compared to verification items. RC condition (*p* = .919) and interaction (*p* = .672) were not significant.

*Re-reading Time*. No significance was found for RC condition (*p* = .554), task type (*p* = .917) and interaction (*p* = .944) during re-rereading time at the adverb.

*Go-past Time*. In contrast with first-pass RT, while task type (*p* < .001) was significant, verification sentences had longer go-past RTs compared to plausibility sentences. Once again, RC condition (*p* = .246) and interaction (*p* = .065) were not significant.

*Regression-out*. Regression-out revealed the same findings as go-past. Task type (*p* < .001) demonstrated that within the verification task there was a higher likelihood of regressing back. Neither RC condition (*p* = .704) nor interaction (*p* = .316) were significant.

*Regression-in*. RC condition (*p* = .184), task type (*p* = .696) and interaction (*p* = .347) were not significant.

#### Matrix verb (V2)

*First-pass RT*. It was shown that for task type (*p* < .01), plausibility sentences initially had significantly longer first-pass RTs than verification sentences. Neither RC condition (*p* = .450) nor interaction (*p* = .083) were significant.

*Re-reading Time*. Similar with first-pass, task type (*p* < .05) revealed longer re-reading times for the plausibility task. Once more, RC condition (*p* = .678) and interaction (*p* = .115) were not significant.

*Regression-out*. While task type (*p* < .001) was significant, it was found that opposite to first-pass and re-reading, within the verification task there was a higher chance of a regression occurring out of the verb. RC condition (*p* = .206) and interaction (*p* = .621) were not significant.

In the following sections we include additional analyses separate from the main findings to give further insight on how these sentences were processed. As discussed above, the word/thematic order is a confounding factor in temporarily ambiguous contexts. ORCs are facilitated by the surface canonical SVO word and agent-to-patient order in Mandarin while VOS word and patient-to-agent ordered SRCs deviate from it. Accordingly, the RC structure (N1, V1, DE) would be predicted to be easier to process on the basis that canonicity would support ORCs before the head noun since the relativizer satisfies the categorical selectional restriction of the RC verb. Additionally, we included the matrix clause (N2, ADV, V2) to widen the scope to include associated effects of the matrix subject and matrix verb together.

#### Full RC structure (N1, V1, DE)

*First-pass RT*. For the first reading of this region, RC condition (*p* < .01) demonstrated that SRCs had significantly longer first-pass RTs compared to ORCs. Also, task type (*p* < .001) showed that within the verification task, first-pass RT was significantly longer than within the plausibility task. Interaction (*p* = .085), however, was not significant.

*Re-reading Time*. For the re-reading of this expanded RC region, RC condition (*p* = .354) was not significant. Task type (*p* < .001) showed that significantly longer RTs were required for the verification task. The interaction of RC condition and task type, however, was significant (*p* < .05). This interaction revealed that within the plausibility task (*p* = .365), ORC:Plausibility did not have significantly different RTs compared with SRC:Plausibility. On the other hand, in the verification task (*p* < .05) SRC:Verification had significantly longer re-reading times compared with ORC:Plausibility.

*Regression-in*. For regression-in proportion, task type (*p* < .001) demonstrated that verification sentences had a higher probability of having a regression back into the RC compared with plausibility task sentences. RC condition (*p* = .758) and interaction (*p* = .096) were not significant.

#### Matrix clause (regions N2, ADV, V2)

*First-pass RT*. For the first reading of the matrix clause region, RC condition (*p* < .01) revealed that ORCs had significantly longer first-pass RTs compared to SRCs. Also, task type (*p* < .001) demonstrated that the plausibility task had longer RTs than the verification task. Interaction (*p* = .575) was not significant.

*Re-reading Time*. There was no effect of RC condition (*p* = .179) during re-reading. Task type (*p* < .001), on the other hand, now revealed that the verification task required longer re-reading times for the matrix clause as a whole. Interaction (*p* = .477) was not significant.

*Regression-out*. Only task type (*p* < .001) was significant, revealing a greater probability of regressing back into the RC for the verification task. Neither RC condition (*p* = .896) nor interaction was significant (*p* = .142).

### Discussion

The results of Experiment 1 for both tasks revealed a general pattern of SRC difficulty within the relative clause and ORC difficulty at the main clause. SRC difficulty was indicated by the increased go-past time and regression-out proportion at the relativizer and head noun, as well as the increased first-pass RTs for the expanded RC structure. In contrast, ORC difficulty was seen primarily during the first-pass reading of the matrix clause and for the regression-in proportion at the head noun.

Despite the fact that both tasks produced relatively similar results, RTs differed between the two tasks; specifically, RTs increased within the verification task for the large majority of the measures. Also, the initial reading of the RC phrase (N1, V1) during first-pass RT was significantly longer for ORC:Verification in comparison to SRC:Verification while ORC:Plausibility was faster, yet not significantly so, compared to SRC:Plausibility. This discrepancy between tasks may possibly be attributed to the participants reading more slowly initially within the verification task at this region. We suspect two possibilities for this: (1) a pro-drop interpretation may have been initially considered and appeared more natural for participants, or (2) the longer reading times at this region allowed participants to reject the matrix clause interpretation prior to reading the relativizer thus initially supporting expectation-based processing. Caplan et al. [50] argued that for verification judgments the differences between tasks may be due to a strategy involving the repeated rehearsal of the sentence, during its display, in order to answer the post-sentence question. As such, we believe the increased reading times for the verification task compared to the plausibility task likely reflected a task strategy where participants slowed down their reading of the sentence for this purpose. Despite the difference in overall RTs, the general pattern of results (e.g., an ORC advantage within the RC structure and a disadvantage within the matrix clause) was seen in both tasks with only minor differences. Accordingly, we believe that the main findings are not task artefacts and that both tasks tapped into RC processing in a similar fashion.

The overall findings provided clear evidence of SRC difficulty at the relativizer, head noun, and relative clause structure (i.e., N1, V1, DE). These results appear consistent with previous eye-tracking and ERP studies showing difficultly for SRCs at the relativizer and head noun within ambiguous RCs. Furthermore, the results for the RC structure as a whole are compatible with the combined response times for a similar combined RC region found in Qiao et al.’s [25] maze task. In general, these results are compatible with models that support an ORC advantage: (i) they generate fewer predicted syntactic heads in storage, (ii) the expectations made on the incorrect matrix clause interpretation can facilitate the reading within the ORC, and (iii) ORC heads are easier to integrate with the gap due to linear/temporal-based integration locality. Packard et al.’s [23] assertion that the relativizer can serve as a potential filler during integration was found to be supported by the increased first-pass RT, go-past RT and increased likelihood of regressing out at the relativizer for the SRC condition.

While observing RTs at individual regions, there was little evidence (e.g., regression-in at the head for the plausibility task) to suggest a similarity-based interference. However, when viewing the entire matrix clause, ORC processing difficulty was observed in both tasks, as indicated by the significantly faster first-pass RTs for SRCs. Considering that this difficulty for ORCs seems associated with the processing of the matrix verb, we feel that these results could hint at a similarity-based interference.

Additionally, these results at the matrix clause may also provide some support for accounts on animacy preferences in Mandarin. In short, since ORCs are less frequently found to be animate-animate compared to SRCs, this preference could manifest itself in a slowdown in reading within the matrix clause. While we did not test for animacy in this study, we cannot rule out completely that animacy had some effect making ORCs more difficult at these loci since animacy effects during parsing have been well documented [60,61].

The results of Experiment 1, however, are not compatible with proposals supporting ORC difficulty based on expectation-based processing for the RC itself, save the initial first-pass reading of the RC for the verification task. The differences between past studies and our own possibly originate from the fact that Experiment 1 used both ambiguous RCs and eye-tracking. Previous studies finding ORC difficulty either used unambiguous RCs and eye-tracking or used ambiguous RCs in a moving window design. Considering that eye-tracking allows for “normal” reading, while moving-window paradigms do not, there may be differences in the degree of sensitivity for each method. As previously mentioned, ambiguous items are highly confounded by Mandarin’s canonical order. Considering this, items in Experiment 2 were based on Jäger et al. [17] in order to test for the effect of the initial ambiguity on RC processing. Accordingly, Experiment 2 will test if the above findings are indicative of a simple garden path effect or if the results reflect a more intricate pattern of processing involving multiple processing factors. It is our opinion that it is the latter and that multiple factors may be playing a role: canonicity, memory-based constraints, and expectation effects.

## Experiment 2

The purpose of Experiment 2 is to determine whether the ambiguity of the RC alters the processing of Mandarin RCs. More specifically, we question whether the above results reflect a simple garden path effect due to the canonical word order of ORCs within ambiguous contexts or if canonicity and linear/temporal locality are also applicable under a less ambiguous context. We also investigate if expectation-based processing is the dominant factor guiding processing of unambiguous RCs. Lastly, we aim to verify our claim that similarity-based interference may be responsible for increasing ORC reading times within the matrix clause region.

## Materials and methods

### Participants

Forty-one native speakers of Mandarin Chinese, all from Mainland China, were recruited from Nagoya University in Japan. Four participants were removed due to calibration errors (leaving N = 37; Female = 27). The mean age of the participants was 25.2 years (range 22–33 years). None of these participants took part in Experiment 1.

#### Materials

The items for Experiment 2 were analogous to the eye-tracking items and questions of Jäger et al. [17] (which, in turn, originated from Gibson and Wu [23]). Considering these items were designed for Taiwanese speakers of Mandarin and not Mainland Chinese speakers of Mandarin, minor modifications to the text were required to better suit the intended participants of this study. These modifications involved converting the script from traditional to simplified Chinese since only mainland Mandarin speakers were recruited. Also, several words and phrases were changed to make them more appropriate and natural for mainland Mandarin speakers. Specifically, 13 of the 32 items contained modifications; out of those 13, six items had their frequency phrase (see below) replaced with another frequency phrase found in other stimuli items. While Jäger et al. [17] used both subject- and object-modified relative clauses, only subject-modified relative clauses were used in the current study. This allowed us to keep the number of items the same per condition between studies. In addition, object-modified RCs were also not included since in situ object-modified RCs are not preferred in Mandarin. In situ object-modified RCs are instead preferred to be topicalized to the front of the sentence [3]; see below for the item conditions.

The items from Jäger et al. [17] were designed to have two syntactic cues which would be able to help attenuate the initial ambiguity: (i) a sentence initial determiner and classifier (henceforth “Det+Cl”) for increased head noun anticipation at the start of the RC, and (ii) a frequency phrase adjacent to the relativizer to provide an increased chance for an RC interpretation prior to the relativizer. The initial Det+Cl inserted prior to the RC was followed directly by a temporal adverb which could not be modified by Det+Cl. In a sentence completion task, they [17] found that interpretations of a missing pronominal intervening between the two phrases was only taken 10% of the time for SRCs and never for ORCs. Accordingly, the combination of the two phrases keeps Det+Cl open for modifying another noun in the sentence. Furthermore, the temporal adverb prevents modification of the Det+Cl with anything within the RC therefore leaving it open for the head noun. Consequently, Det+Cl acts as a syntactic cue to help eliminate the matrix clause interpretation for the RC as well as increasing anticipation for the noun modified by it. In the current experiment, we manipulated the subject-modified RCs to either have the initial Det+Cl present (i.e., reducing the level of ambiguity, henceforth “DCL”) or omitted (i.e., ambiguous, henceforth “Empty”). The frequency phrase was present in all items. It is important to note that the position of the frequency phrase for ORCs is not natural and would appear ungrammatical within a matrix clause. For both ORCs and SRCs, the frequency phrase was implemented to prevent the relativizer from being interpreted as a genitive marker. Thus, its inclusion enhances an RC interpretation at the relativizer locus.

We used a 2 (RC condition: ORC vs. SRC) x 2 (determiner type: Empty vs. DCL) design for the 32 experimental items. In the example below, Det+Cl stands for the Det+Cl modifying the head noun, ADV is a temporal adverb for the RC, V1 is the embedded RC verb, N1 is the RC noun, Freq is the frequency phrase, DE is the relativizer, N2 is the head noun, V2 is the first matrix verb, and N3 is the first matrix object. The remainder of the sentence is not denoted. Since the verification task was repeated in Experiment 2, an equal number of true and false verification/comprehension probes were given per counterbalanced list. See the Appendix for a list of all experimental stimuli.

SRC / ORC items of Experiment 2

(Det+Cl) ADV        V1        N1      /      N1          V1        Freq DE

(*Nàgè*) [*Zuówǎn t*_i_ *Zòule Fúwùshēng* / *Fúwùshēng Zòule  t*_i_        *Yīdùn        De*]

that.one [last.night *t*_i_ beat    waiter    /    waiter beat    *t*_i_  more.than.once REL]

          N2        V2        N3

        *Gùkè*_i_    *Tīngshuōguò    Lǎobǎn    Bìngqiě    Jìdé    Tā*

        *customer*_i_  heard.of    boss        and    remember him

      ‘(The) customer [who beat up the waiter / the waiter beat up last night] has heard of the boss and remembers him.’

#### Procedure

The procedure was similar to Experiment 1. All characters were displayed in simplified Chinese SimSun 22pt font, a visual angle of 1.8°. The font and size were changed to better fit the longer stimuli used in Experiment 2. Here, participants now had a maximum of 12 seconds to read the sentence and press any button when they were finished reading to replace the sentence with the question. Participants still had a maximum time of eight seconds to answer the verification/comprehension probe. The increase in allotted time also accommodated for the increased length of the items.

### Results

**Fig 4:**
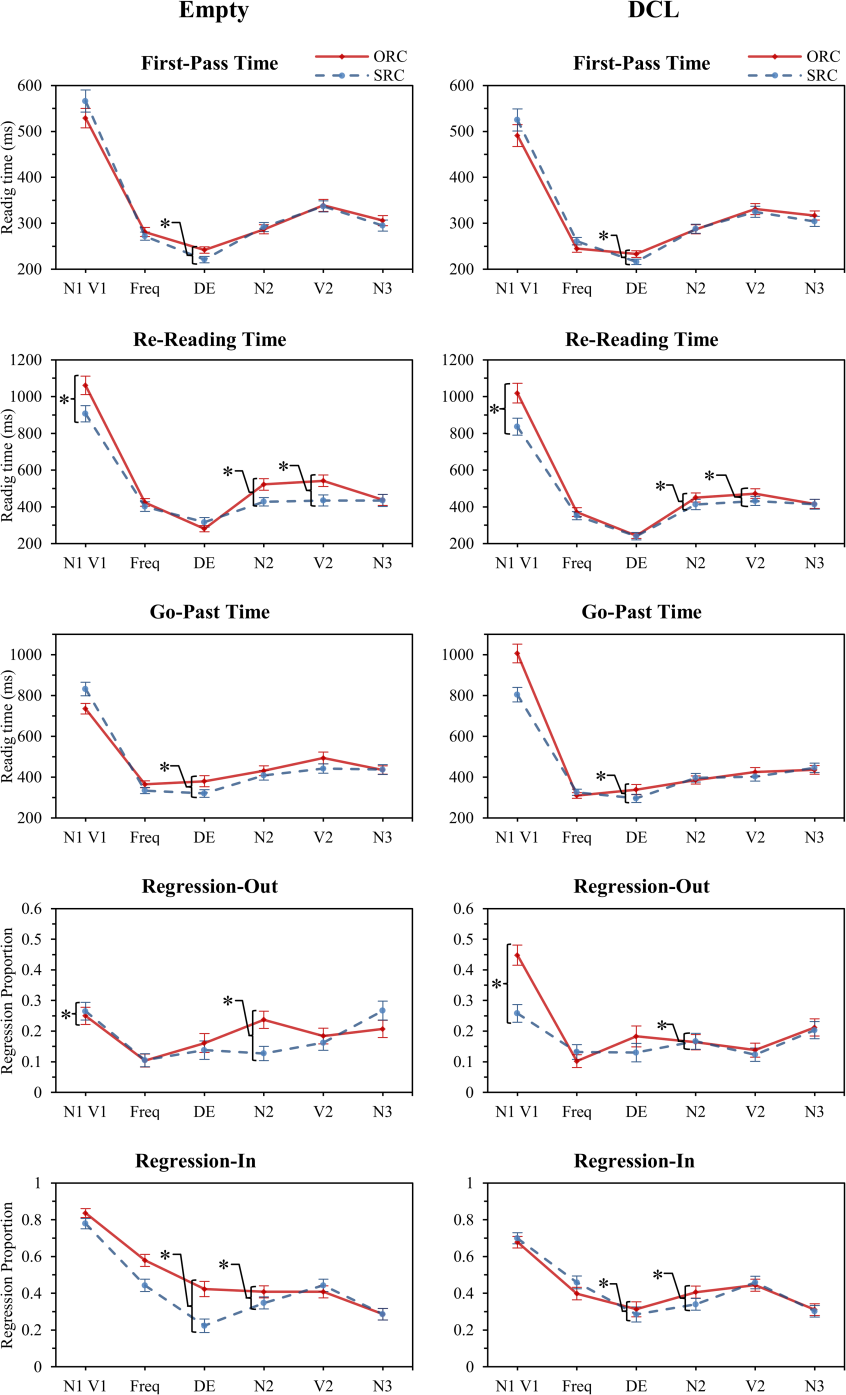
Experiment 2: The trimmed reading times and regression proportions.

Eye-fixations were treated following the same procedure as Experiment 1 which resulted in the removal of 1,963 fixations or 7.34%. The same LME methods were used as in Experiment 1. RC condition (ORC vs. SRC) and determiner type (Empty vs. DCL) were considered as fixed effects, and subject and item composed the random effects. If interaction of condition:type was significant, a pairwise analysis was conducted. Data trimming for each model resulted in the removal of 1.68% of the data. Refer to Tables D-H for means and standard errors and LME results within S1 Tables. Following Jäger et al. [17], we analysed N1/V1 (RC), Freq (frequency phrase), DE (relativizer), N2 (head noun), V2 (matrix verb) and N3 (matrix object). We also analysed the sentence as a whole (accuracy and total reading time), the RC structure (N1, V1, Freq, DE) and matrix clause (N2, V2, N3) as in Experiment 1. The trimmed reading times, regression proportions and fixed effect significance for RC condition per determiner type (Empty and DCL share RC condition fixed effect significance), individual region and eye-tracking measure are shown in Fig 4.

#### Sentence

*Accuracy*. The analysis on the accuracy for the verification probes revealed no significant differences for RC condition (*p* = .516), determiner type (*p* = .920) or condition:type interaction (*p* = .531). The mean scores were rather close between items.

*Total reading time of the sentence*. For the reading of the sentence, while both RC condition (*p* < .001) and determiner type (*p* < .01) were significant, interaction was not (*p* = .800). The general pattern of results revealed that the ORC condition had longer RTs than the SRC condition and that the DCL type had longer RTs compared to the Empty type.

#### RC (N1, V1 / V1, N1)

*First-pass RT*. For the RC condition (*p* = .068), even though ORCs were read quicker than SRCs, the result was not significant. For determiner type (*p* < .001), the Empty type had significantly longer RTs than the DCL type. Interaction was not significant (*p* = .428).

*Re-reading Time*. In contrast to first-pass RT, ORC re-reading time was significantly longer than SRC re-reading time at this later stage of processing (*p* < .001). For determiner type, while the Empty type had longer RTs in comparison to the DCL type, the difference did not reach significance (*p* = .063). There was still no effect of interaction (*p* = .504).

*Go-past Time*. While there was no significant difference between RC conditions (*p* = .129), there was a significant difference in determiner type (*p* < .05) showing unsurprisingly that the DCL type had longer RTs than the Empty type since the DCL type items had one additional region compared with the Empty type items. During this stage, there was a significant effect of interaction (*p* < .001). The pairwise comparison revealed that ORC:DCL had significantly longer RTs than SRC:DCL (*p* < .001). While ORC:Empty had the lowest RTs, it was not significantly faster than SRC:Empty in the pairwise analysis.

*Regression-out*. The RC condition (*p* < .01) and determiner type (*p* < .01) revealed that ORCs were more likely to have a regression out than SRCs, and the DCL type was more likely than the Empty type. Again, there was a significant effect interaction showing that ORC:DCL was more likely to regress out than SRC:DCL (*p* < .001). Consequently, it appears that ORC:DCL was driving the effects for this measure.

*Regression-in*. While RC condition (*p* = .299) was not significant, determiner type (*p* < .001) demonstrated that the Empty type was more likely to have a regression made back into the RC in comparison to the DCL type. Interaction was not significant (*p* = .141).

#### Frequency phrase (Freq)

*First-pass RT*. At the first-pass reading of the frequency phrase, there were no differences between RC conditions (*p* = .179), but determiner type (*p* < .05) demonstrated that the Empty type had longer RTs compared to the DCL type. Interaction was not significant (*p* = .075).

*Re-reading Time*. RC condition (*p* = .146) and interaction (*p* = .368) did not show significant differences during re-reading. Again, determiner type (*p* < .05) revealed that the Empty type had significantly longer RTs compared to the DCL type sentences.

*Go-past Time*. RC condition (*p* = .844) was still not significant during go-past time, while determiner type (*p* < .05) still demonstrated that the Empty type had longer RTs compared to the DCL type. Interaction of condition:type was significant (*p* < .05). However, this only demonstrated that ORC:Empty had significantly longer go-past RTs than ORC:DCL (*p* < .01).

*Regression-out*. RC condition (*p* = .514), determiner type (*p* = .680) and interaction (*p* = .540) revealed no significant differences.

*Regression-in*. RC condition (*p* = .131) was not significant, but determiner type (*p* < .05) revealed that the Empty type was more likely to have a regression back into the frequency phrase than the DCL type. There was a significant effect for interaction (*p* < .01), demonstrating that ORC:DCL was less likely to have a regression back into the phrase than SRC:DCL (*p* < .05).

#### Relativizer (DE)

*First-pass RT*. For the RC condition (*p* < .05), it was shown that ORCs had significantly longer RTs than SRCs. Neither determiner type (*p* = .554) nor interaction (*p* = .415) at the relativizer were significant.

*Re-reading Time*. In later re-reading times, the RC condition (*p* = .543) was no longer significant. However, determiner type (*p* < .01) indicated that the Empty type had longer RTs than the DCL type. Interaction was not significant (*p* = .311).

*Go-past Time*. Only the RC condition (*p* < .05) revealed a significant difference in RTs, showing that ORCs as a whole had longer RTs in comparison to SRCs. There was no significance for determiner type (*p* = .103) and interaction (*p* = .640).

*Regression-out*. The RC condition (*p* = .230), determiner type (*p* = .791) and interaction (*p* = .617) did not reveal any significant differences.

*Regression-in*. For the RC condition (*p* < .01), ORCs were significantly more likely to have a regression back into the relativizer than SRCs (*p* < .01). However, determiner type (*p* = .670) was not significant. While there was a significant interaction effect found (*p* < .05), it only indicated that ORC:Empty was more likely to have a regression back into the relativizer than SRC:Empty (*p* < .01), despite both ORCs having higher regression-in means than their SRC counterparts.

#### Head noun (N2)

*First-pass RT*. RC condition (*p* = .497), determiner type (*p* = .578) and interaction (*p* = .778) revealed no significant differences during first-pass reading.

*Re-reading Time*. For fixations made after first-pass, RC condition (*p* < .05) demonstrated that ORCs had longer RTs than SRCs, and determiner type (*p* < .05) revealed that the Empty type had longer RTs compared to DCL type items. Interaction did not show significant differences (*p* = .806).

*Go-past Time*. While the RC condition (*p* = .692) and interaction (*p* = .340) were not significant, the determiner type (*p* < .05) showed that the Empty type items required longer RTs before moving on to the matrix clause verb.

*Regression-out*. The RC condition (*p* < .05) revealed that the ORC condition was significantly more likely to make a regression out of the head noun back into previous parts of the sentence in comparison to SRCs. Determiner type (*p* = .624) was not significant. However, interaction (*p* < .05) was significant and demonstrated that ORC:Empty was significantly more likely to make a regression out of the head than SRC:Empty (*p* < .05).

*Regression-in*. Only the RC condition (*p* < .05) was significant showing that ORCs were more likely to have a regression back into the head from later parts of the matrix clause. Determiner type (*p* = .911) and interaction (*p* = .938) were not significant.

#### Matrix verb (V2)

*First-pass RT*. RC condition (*p* = .955), determiner type (*p* = .302) and interaction (*p* = .728) revealed no significant differences during first-pass reading.

*Re-reading Time*. For the RC condition (*p* < .01), ORCs had significantly longer RTs than SRCs, whereas determiner type (*p* = .440) was not significant. While interaction (*p* < .05) was significant, the pairwise analysis revealed that ORC:Empty only had significantly longer RTs than SRC:Empty (*p* < .01).

*Go-past Time*. While RC condition (*p* = .209) and interaction (*p* = .967) were not significant, determiner type (*p* < .01) indicated that the Empty type sentences had significantly longer RTs in comparison to DCL sentences.

*Regression-out*. RC condition (*p* = .414) and interaction (*p* = .921) were not significant; determiner type (*p* = .061) also revealed no significance even though the Empty sentences had a higher likelihood to regress out than DCL sentences.

*Regression-in*. RC condition (*p* = .567), determiner type (*p* = .377) and interaction (*p* = .748) revealed no significant effects.

#### Matrix object (N3)

*First-pass RT*. For the RC condition (*p* = .058), no significant differences were found. Also, neither determiner type (*p* = .136) nor interaction (*p* = .417) indicated significant differences during first-pass reading.

*Re-reading Time*. RC condition (*p* = .643), determiner type (*p* = .997) and interaction (*p* = .863) revealed no significant effects.

*Go-past Time*. RC condition (*p* = .562), determiner type (*p* = .921) and interaction (*p* = .377) revealed no significant effects.

*Regression-out*. RC condition (*p* = .405), determiner type (*p* = .264) and interaction (*p* = .295) revealed no significant effects.

*Regression-in*. RC condition (*p* = .912), determiner type (*p* = .537) and interaction (*p* = .886) revealed no significant effects.

Next, we present the additional analyses as described above. Refer to Tables G and H for means, standard errors and LME results in S1 Tables.

#### Full RC structure (N1, V1, Freq, DE)

*First-pass RT*. There was a significant effect for the RC condition (*p* < .01) showing that ORCs were read faster than SRCs. Determiner type (*p* < .001) was also significant and revealed that the Empty type sentences had longer RTs during first-pass reading compared to DCL sentences. Interaction (*p* = .069), however, did not reach the significance threshold.

*Re-reading Time*. The RC condition (*p* < .001) and determiner type (*p* < .05) were both significant which demonstrated that ORC conditions had significantly longer RTs than SRCs and the Empty type items had significantly longer RTs compared to DCL items. Interaction (*p* = .993) was not significant.

*Go-past Time*. The RC condition (*p* = .069) did not reveal a significant difference between ORCs and SRCs. Determiner type (*p* = .151) was also not significant. Interaction (*p* < .001) of condition:type was significant and demonstrated contrasting effects for the ORC types. This interaction showed that the ORC:DCL condition had significantly longer RTs than SRC:DCL (*p* < .001). On the other hand, it was revealed that the ORC:Empty condition had significantly faster RTs than the SRC:Empty (*p* < .05) condition.

*Regression-out*. The RC condition (*p* < .001), determiner type (*p* < .001) and interaction (*p* < .001) were all significant. It was shown that the ORCs conditions and DCL types were significantly more likely to regress out than their counterparts. However, the pairwise analysis indicated that it was only the ORC:DCL condition which was significantly more likely to regress out of the RC structure than SRC:DCL (*p* < .001).

*Regression-in*. While the RC condition (*p* = .097) was unable to reveal significant differences between conditions, determiner type (*p* < .001) and interaction (*p* < .01) were both significant. While the Empty type was significantly more likely to have a regression back into the RC structure, the pairwise analysis revealed that, opposite to regression-out, it was only ORC:Empty which was more likely to have a regression made back into the RC structure in comparison to SRC:Empty (*p* < .01).

#### Matrix clause (N2, V2, N3)

*First-pass RT*. While the RC condition (*p* = .640) and determiner type (*p* = .216) were not significant, interaction (*p* < .01) was significant. However, from the pairwise analysis, it was only revealed that ORC:Empty was significantly faster than SRC:Empty (*p* < .05).

*Re-reading Time*. The RC condition (*p* < .001) revealed that ORCs had significantly longer re-reading times than SRCs, and determiner type (*p* < .01) showed that the Empty sentences had significantly longer RTs compared to DCL sentences. Interaction (*p* = .271) was not significant.

*Go-past Time*. The RC condition (*p* < .01), determiner type (*p* < .001) and interaction (*p* < .01) were all significant. As with re-reading time, ORCs had significantly longer RTs compared to SRCs, and the Empty sentences had significantly longer RTs in comparison to their DCL counterparts. In contrast to first-pass time, the pairwise analysis showed that ORC:Empty now had significantly longer go-past times in comparison to SRC:Empty (*p* < .001).

*Regression-out*. For the RC condition (*p* = .087), ORCs only had a trending likelihood of regressing out of the matrix clause in comparison with SRCs. However, determiner type (*p* < .01) revealed that the Empty type items were significantly more likely to regress out than DCL type items. Interaction (*p* < .01) was significant, and similar to go-past time, the pairwise analysis indicated that ORC:Empty was significantly more likely to have a regression out of the matrix clause in comparison to SRC:Empty (*p* < .001).

*Regression-in*. RC condition (*p* = .661), determiner type (*p* = .088) and interaction (*p* = .942) revealed no significant differences between conditions and types.

### Discussion

In contrast to Experiment 1, Experiment 2 clearly showed that ORCs were more difficult to process than SRCs. Nonetheless, the results also indicated that multiple processing factors were involved in the processing of Mandarin RCs revealing both ORC advantages and disadvantages: Canonicity (ORC facilitation), expectation (ORC disadvantage), and perhaps similarity interference (ORC disadvantage) as well.

While integration resources were not directly supported in Experiment 2, evidence of canonicity was nevertheless present for both unambiguous and ambiguous ORC items during early RTs within both RC regions. Additionally, Jäger et al. (refer to Table 13 in [17]) also appeared to have initial, albeit non-significant, ORC facilitation at the RC (N1,V1) during first-pass reading time. In the current study, however, it was later revealed during go-past RTs at these regions that while ORC:DCL became more difficult in comparison to its SRC:DCL counterpart, ORC:Empty remained easier than its SRC:Empty counterpart. Accordingly, the presence of the determiner increased RTs for ORC:DCL in comparison to SRC:DCL, but just not initially. This initial facilitation for the unambiguous ORC happens to conflict with expectation, canonicity (i.e., that is canonicity models incorporating both frequency and regularity, see [32]) and storage-based models of processing. Expectation-based processing was not supported because ORCs are less frequent and thus should be initially more difficult. While canonicity (i.e., frequency and regularity) appeared to be supported, it is likely the case that it was not since the Det+Cl phrase should have attenuated the simple matrix clause interpretation. In other words, the garden path argument seems no longer valid since there should not have been an initial misparse confusing the RC as a matrix clause. For the ambiguous items lacking the Det+Cl, however, a garden path effect may still have been present which allowed the ORC:Empty items to remain easier to process than SRC:Empty items at the RC structure. This likely suggests that canonicity was influencing processing in a different manner for the unambiguous RCs. Simply put, if an argument is closer to the canonical order, be it grammatical word order or thematic order, facilitation can be predicted regardless of the structure’s actual statistical frequency (here, ORCs are less frequent than SRCs). That is not to say that frequency effects are not important for canonical facilitation, but to instead suggest that in rare contexts where the matrix clause interpretation is no longer attainable, regularity alone may provide facilitation in reading. It may be the case that while a matrix clause interpretation was attenuated, the RC interpretation was only formed after reading the relativizer which allowed the regularities of a simple matrix clause structure to facilitate reading inside the embedded clause.

In addition to canonicity effects, the initial benefit for ORCs may also loosely provide indirect support for linear/temporal metrics of integration. However, integration was not directly supported at the relativizer or head noun which we attribute to antilocality effects. In other words, with the introduction of syntactic cues (e.g., the Det+Cl phrase and the frequency phrase for both sets of items), there would be greater expectation or anticipation [62] for the SRC relativizer and the head in comparison to the items used in Experiment 1 since both syntactic cues favour SRCs.

For expectation-based effects, the general pattern of results observed in Jäger et al. [17] was replicated such that ORC difficulty was not initially seen at the RC until later reading times. In addition to these results, there was also an influence of ambiguity. ORC:DCL became more difficult to process than its SRC counterpart earlier compared to ORC:Empty in respect with its SRC counterpart. Despite this, surprisal effects were largely supported at the relativizer where both ORCs had increased RTs in respect to their SRC counterparts. Jäger et al. [17], however, did not reveal an effect of surprisal at the relativizer. While Experiment 1 and other studies revealed an opposite trend at the relativizer, the observation of late ORC difficulty within the RC can be partially attributed to the presence of the frequency phrase (Freq), which helps provide the RC with its correct interpretation. In turn, the cue likely increased expectation for the relativizer within the RC conceivably causing an antilocality effect at both loci of integration (i.e., the relativizer and head noun). What is more, the position of the frequency phrase is not in a natural position for ORCs which may make the phrase appear initially ungrammatical without Det+Cl. However, no significant differences were seen between determiner types during early measures. At the very least, the frequency phrase may have partially contributed to the ORC difficulty found at the relativizer and head noun.

Similarity-based interference was again hinted at by the indication of ORC difficulty at the matrix clause. Since Jäger et al. [17] also found significantly longer total reading time at the matrix verb for subject-modified ORCs using eye-tracking, we suspect the similarity interference effect here is relatively minor, but nevertheless present. In Experiment 2, the Empty conditions had increased RTs compared to DCL counterparts during later measures. As such, the presence of Det+Cl may have made the DCL items less susceptible to interference from the RC noun. Considering these points, we believe that this finding is better representative of similarity interference rather than the influence of animacy. Animacy, however, still cannot be completely ruled out as a contributing factor.

In summary, while canonicity facilitated ORCs early on with indirect support for linear/temporal integration, the influences of expectation-based processing later reversed this within the RC. At the matrix clause, similarity-based interference was also observed to be a potential factor responsible for increasing ORC difficulty. In all, the reading of these sentences was seen to be influenced by multiple factors of processing.

## General discussion

In this study, we sought out to determine which Mandarin relative clause structures are more demanding to process. We investigated the reading of ambiguous RCs as well as unambiguous RCs using eye-tracking. More specifically, we aimed to determine how the initial clause type ambiguity and processing factors such as canonicity, expectation, integration and similarity-based interference influence the reading of Mandarin sentences containing RCs. The results of Experiment 1 revealed that ambiguous ORCs were generally easier to process than SRCs, regardless of task design supporting canonicity, expectation, storage and integration-based effects. Yet, in the long run, ORCs became more difficult to process at the matrix clause, a result which may provide support for similarity-based interference as well as accounts on animacy preferences in Mandarin RC processing. The results of Experiment 2 revealed that canonicity and possibly locality facilitated the early readings of the ORC within the relative clause. Also, ambiguous ORCs remained easier to process compared to SRCs longer than unambiguous ORCs. ORCs were still more difficult during later RTs within the RC and matrix clause as explained by expectation-based processing and similarity interference. Experiment 2, however, did not provide direct evidence supporting linear/temporal integration-based models at the relativizer or head noun. This was possibly due to antilocality effects or due to the inclusion of the frequency phrase in items used in Experiment 2, given the irregular position of the phrase for ORCs.

One particular framework of processing and cognitive behaviour can support the findings of this study, that is, Lewis and Vasishth’s [36,38] activation-based model within the scope of ACT-R. Vasishth and Lewis [38] consider both bottom-up and top-down mechanisms to have corresponding interdependent influences on the activation level of a particular node in the sentence structure. Lewis and Vasishth [36,38] note three constraints for activation levels: (1) locality, (2) anticipation, and (3) similarity interference. Here, we would like to add an additional and interactive constraint, (4) canonicity, which has often been shown to support processing and comprehension across languages such as Basque [63], German [64], and Japanese [65,66]. As Love and Swinney [67] suggested, however, languages may differ in how (and if) they benefit from canonicity. Put another way, the influence of canonicity may fall along a continuum across different languages.

We view canonicity as a top-down mechanism based upon a coarsely-tuned account of a language’s structural or thematic regularities. While expectation and anticipatory effects may be more dependent on fine-tuned structural and collocational frequencies, canonicity can influence processing even for less frequent structures based solely on regularities of the language. This interpretation would therefore differ from and supplement previous notions of canonicity which have been based upon both statistical frequency and regularity [32]. We find that this additional interpretation of canonicity, separate from storage-based and expectation-based processing, provides the best interpretation as to why unambiguous ORCs were initially read more quickly. In other words, despite ORCs being less frequent not only in overall structure but also after a Det+Cl phrase, ORCs nevertheless received some benefit from their relationship to the canonical word or thematic order of Mandarin. In contrast, a storage-based or an expectation-based account would predict initial ORC difficulty instead of SRC difficulty for these items if a matrix clause interpretation was attenuated. Considering that Experiment 1 used ambiguous RCs and did not contain any syntactic cues to hint at an RC interpretation, the combined influences of canonicity, locality and possibly storage-based resources likely impacted the processing of the ORC phrase much more than the expectation for the SRCs at the relativizer. Recall that Mandarin Chinese is rather unique in being a right-branching language that displays left-branching prenominal RCs, and that the less frequent ORC structure follows the canonical SVO and agent-to-patient word and thematic orders. Following this, the effect of canonicity against expectation effects may be exclusive to languages such as Mandarin Chinese displaying this infrequent language pattern. Specifically for Mandarin RC processing, we believe this influence of canonicity is best observed globally for the RC structure as a whole whereas expectation-based processing, such as surprisal, is more localized at individual regions.

In contrast with canonicity, as syntactic cues which helped give an RC interpretation were introduced into the sentence (e.g., the ambiguous Empty types and unambiguous DCL types in Experiment 2), anticipatory processes greatly influenced the processing for the more frequent SRC structure. This caused SRCs to be processed more easily than ORCs at the relativizer and during later reading times for the RC (N1, V1) and RC structure (N1, V1, Freq, DE) in Experiment 2. However, we understand this greater expectation or anticipation for the SRC structure to be an antilocality effect. We believe this effect could have possibly prevented the observation of a linear or temporally defined integration metric at the relativizer and head noun. As mentioned above, locality is a constraint on the reactivation of an item from memory. In general, after the initial activation of an item, the activation level will begin to decay, and the more distant a gap is to its filler, the greater the decay will be. Since ORCs would have less activation decay due to the gap and filler being more local defined by either a linear or temporal metric, ORCs should be easier to process when integrating filler-gap dependencies. This was clearly supported by the results of Experiment 1. Experiment 2, on the other hand, only was able to support effects of locality beyond the scope of the specific loci of integration in Mandarin Chinese. If we consider that locality does influence processing, then the fact that the results of Experiment 2 conflict with ORC locality is best explained by antilocality effects, rather than a structural-phrase integration metric. Lastly, there was partial evidence supporting a similarity-based interference when the matrix verb needed to retrieve its subject (i.e., the head noun) from memory. This was indicated by the ORC difficulty found at the matrix clause for both experiments and all ORC types. We believe that similarity-based interference provides the most suitable explanation for the ORC difficulty here. The difficulty for ORCs at the matrix clause verb can be explained by the proactive interference of the ORC relative clause subject on the activation level of the matrix clause subject. On the other hand, the SRC relative clause object should not lower the activation level of the matrix clause subject. Thus, during the retrieval of the subject at the matrix verb, ORCs should have greater processing difficulty compared to SRCs.

In summary, the results seem to be compatible with activation-based constraints on processing showing multiple influences on sentence processing. In the current study, we limited these to more global interpretations on sentence processing; as such, see Vasishth and Lewis [36,38] and citations within for a more detailed account for these activation constraints.

### Issues to address

The current study is not without limitation and there are several issues left to be addressed. Both experiments potentially involved issues since animacy, passivation and object-modification were not addressed as independent factors. Consequently, the current study is somewhat limited in its overall interpretability. One issue, for example, is that the current study cannot dissociate semantics and syntax for canonical order effects. Yet, considering that ORCs are preferred to include the passive marker, the thematic canonicity of agent-to-patient may admittedly have a greater influence on processing compared to grammatical SVO word order.

In the current study, while subject/object asymmetry was only investigated for RC processing in Mandarin, subject biases have also been observed within other structures as well. For instance, Simpson, Wu, and Li [68] using a sentence completion task revealed that for pronoun anaphora resolution in Mandarin there was a general preference to form an antecedent relationship with the subject of a preceding sentence. This result was also supported by corpus data which revealed that subjects are predominately found to be the antecedent of a pronoun. Seeing that there is a general tendency to form an antecedent relationship with the subject of a clause, be it embedded or matrix, it may be worthwhile for future studies to also investigate pronoun anaphora in Mandarin to further detail the interrelationship of memory-based and expectation-based models of processing. Furthermore, Simpson et al. [68] found that by altering the coherence relation of the prompt used for the sentence completion task, the number of subject antecedents was increased or reduced. Considering the influence of discourse semantics on pronoun anaphora in Mandarin, future studies can adopt similar experimental methods as Simpson et al. [68] for RCs in Mandarin to tease apart the effects of syntax and semantics on RC processing.

Concerning canonical order facilitation, while the current study found clear benefits of canonicity at the RC structure for both experiments, it is still unclear what role statistical frequency can be attributed to for the items with attenuated clause type ambiguity. Hsiao and MacDonald [10] found that for the statistical regularity of ambiguous RCs and competitor interpretations in Mandarin, numerous interacting factors (e.g., animacy, RC type and modification position) are highly involved in areas of ambiguity. Yet, in the case which the clause type ambiguity is attenuated by the Det+Cl phrase, it is uncertain if competitor interpretations based on simple matrix clauses are permissible; it is our belief that they are likely not. Instead, we assert that while rejecting the matrix clause interpretation, it is conceivable that a RC interpretation was not yet committed. Therefore, the regularities of the word or thematic order could facilitate the clause despite not being garden pathed. As they argue and we certainly agree with, ORC advantages and disadvantages are highly dependent on the context in which they are found. Consequently, further investigation may be needed to clarify which statistical regularities are being utilized for the initial processing of unambiguous relative clauses and if these regularities are counter or congruent with the interpretations made upon the structure.

A notable issue of this study was that the frequency phrase in Experiment 2 still acted as a syntactic cue to help attenuate ambiguity. Thus, the items lacking the Det+Cl were still less ambiguous than the items of Experiment 1. Furthermore, the position of the frequency phrase is unnatural for the ORC condition. Consequently, the difficulty found at the relative clause or head noun for ORCs during later RTs may be attributed in some part to the unnaturalness of the frequency phrase for ORCs. Since the phrase is not in a canonical position for ORCs, it may also be the case that semantics rather than word order may have been facilitating ORCs during early RTs at the full RC region. Future studies using eye-tracking should further address the issue of semantics and also address the frequency phrase as experimental factors to determine its influence inside the RC and at head noun.

In a similar vein, since the Det+Cl can either appear prior or after the RC, it may be best to compare such a design to determine the influence of modification position on the processing of the head noun using eye-tracking. In fact, previous research [69] has already shown that pre-RC classifiers occur predominately in both subject- and object-modified expressions for SRCs whereas ORCs prefer to have post-RC classifiers. It was shown [69] that for pre-RC classifiers, SRCs received a greater benefit from the cue. Accordingly, it was not surprising SRCs were ultimately easier than ORCs in the current study, considering these past findings. In conjunction with the frequency phrase, we believe that these combined disadvantages for ORCs in the item design attributed to the antilocality effect at the relativizer and head noun.

An additional issue was that object-modified RCs were not addressed in this study. Considering that in situ object-modified RCs are not preferred, we believe future studies should follow Lin and Garnsey [70] and investigate object-modified RCs in a topicalized position instead of placing RCs at the in situ position where they would be prone to garden path [71] and clause boundary effects.

There are several other possible issues in the items used as well. Since 13 items were slightly modified, a post-hoc naturalness decision task was carried out on all the RC items to ensure that the 13 modified items did not differ in naturalness from the 19 unmodified items. For this, ten native speakers of Mandarin (female = 10; age range: 22–33 years) volunteered to rate the stimuli on a 1–5 scale Likert scale at Nagoya University in Japan. All volunteers originated from Mainland China and none participated in either eye-tracking experiment. A LME model was used to investigate this difference. RC condition (ORC vs. SRC) and item modification (modified vs. unmodified) were the fixed effects (each coded as -.5 and .5 respectively), and items and subjects were included as random intercepts and slopes as determined by model comparisons. The naturalness rating was coded from -2 (unnatural) to +2 (natural). The result of the analysis revealed that there while was a significant effect of RC condition [coef. = 0.89, SE = 0.17, *t* = 5.14, *p* < .001], neither item modification [coef. = 0.17, SE = 0.20, *t* = 0.87, *p* = .389] nor the interaction of the two [coef. = -0.03, SE = 0.24, *t* = -0.11, *p* = .911] were significantly different. It was found for both the modified and unmodified items, SRCs (Mean = 0.84, SE = .05) were rated significantly higher than ORCs (Mean = -0.05, SE = .06). In Jäger et al. [17], it was found that there was also a numerical difference showing higher acceptability for SRCs but was found to be not significant. The likely difference between the current study and theirs [17] could likely reflect random variability from participant judgements. As such, we assert that the modified items used in Experiment 2 of the current study should not be considered any less or more natural than the items from Jäger et al. [17]. Another possible issue in the items used is that one particular item may have given an undesired interpretation. The RC noun of item 19 from Experiment 2 (see the Appendix below) is *zuòjiā* ‘foreign ministry’. This particular noun may possibly be considered as a location rather an agent for the ORC condition. However, since this item was not modified from the previous study [17] and the overall of pattern of results did not change with its exclusion, we decided to not remove the item from the analyses.

The last limitation addressed here is that relatively few participants were recruited in both experiments and with the high number of analyses conducted in the study, the possibility remains that Type S and M errors were obtained leading to results favouring both ORCs and SRCs within both experiments [72]. However, considering that the overall results of Experiment 1 and 2 replicated previous findings, we do not believe our findings to be overly spurious or misrepresentative, if such errors exist within our analyses.

#### Random variability

Another interpretation besides fluctuations in activation is that expectation or memory effects are not always visible in reading time data. Consequently, self-paced reading studies, complemented with eye-tracking and ERP studies, seem to produce opposing results between studies. Though Vasishth et al. [20, pp. 10–12] argued for an overall SRC advantage, they allowed that random variability may possibly contribute to this appropriate inconsistency to some extent. If there is random variability, then the possible contributing factors should be determined. It is possible that differences in experimental items, the method or the number of cues to attenuate temporary ambiguity, the experimental methodology (i.e., self-paced reading, maze task, eye-tracking, & ERP), and participant-pools (e.g., dialect and exposure to other languages) may all contribute to the random variability. For instance, there have been many studies using Gibson and Wu’s (27) items and unambiguous design [17,20,28], but even among them and the current study, there are inconsistencies in the findings between studies. Specifically, the current study and Jäger et al. [17] have diverging results at the relativizer region for subject-modified RCs.

Although the previous studies and the current study used native Mandarin speakers (with the majority recruiting participants originating from either Mainland China or Taiwan), there is still variability among regional dialects of Mandarin. For instance, even among similar Pǔtōnghuà and Guóyǔ standard dialects of Mandarin (i.e., Standard Mainland China Mandarin and Taiwanese Mandarin), there are differences in grammar, phonology, and vocabulary. As such, it may be of empirical interest for future studies to assess the influence of dialect.

## Conclusion

In an effort to further previous eye-tracking studies that used either ambiguous relative clauses in Mandarin or syntactic cues to attenuate ambiguity, the current study shows that canonicity and linear/ temporal-based integrations metrics support an ORC advantage. However, these effects are more prominent when the structure of the RC is initially ambiguous. As such, we also show that as additional syntactic cues are given, the more likely, quickly and severely the expectations generated from the structural frequency will impact the processing of object-relative clauses. We view this as an antilocality effect. In addition, we also show evidence for a similarity-based interference within the matrix clause regardless of ambiguity. We argue along the lines of Vasishth and Lewis [36,38] that multiple processing factors (e.g., locality, anticipation, expectation, similarity interference and canonicity) constrain the activation level of items and more work is needed to detail their relationships within sentence processing. Consequently, we assert that for Mandarin Chinese, relative clause processing should not be viewed under the scope of a single model or context but rather under an interdependent model.

## Ethics statement

At Nagoya University, Japan ethic committees are operated separately from the main institution within each graduate school; however, not all graduate schools have a committee. Since the Graduate School of Languages and Cultures at Nagoya University, Japan did not have an ethics committee at the time of the study, approval from such a committee could not be obtained. Instead, this research was approved by the faculty of the Graduate School of Languages and Cultures at Nagoya University, Japan which adheres to the Declaration of Helsinki for research using human subjects. In the current study, all participants first signed the informed written consent form prior to participating in the study and received monetary compensation at the end of their session. All personal information collected from participants was stored in a secured location, and participants were given pseudonyms for data analysis purposes. Participants were not subject to harm and could only experience mild discomfort from prolong seating and reading. Lastly, we declare that each of the participating authors did not have any conflict of interest during the completion of the study.

## Appendix

The following sentences are all the ORC experimental items from Experiment 1. The SRC sentence condition is given only for the first item. The interest regions are designated between asterisk marks (N1/V1, DE, N2, ADV, V2).

1 ORC: *李芳邀请*的*梁媛*刚才*迟到了*
  Li Fāng yāoqǐng de Liáng Yuán gāngcái chídàole
  Li Fang invited REL Liang Yuan just late
  ‘LiangYuan who LiFang invited was late just now.’
1 SRC: *邀请李芳*的*梁媛*刚才*迟到了*
  yāoqǐng Li Fāng de Liáng Yuán gāngcái chídàole
  invited Li Fang REL Liang Yuan just late
  ‘LiangYuan who invited LiFang was late just now.’
2 *王磊联系*的*张艳*刚才*进门了*
  WángLěi liánxì de Zhāng Yàn gāngcái jìnménle
  Wang Lei contact REL Zhang Yan just entered
  ‘Zhang Yan who Wang Lei contacted just entered the door.’
3 *王艳走访*的*王伟*前天*参赛了*
  Wáng Yàn zǒufǎng de Wáng Wěi qiántiān cānsàile
  Wang Yan visited REL Wang Wei day.before.yesterday entered.competition
  ‘Wang Wei who Wang Yan visited entered the competition the day before yesterday.’
4 *张伟勾结*的*李强*去年*入狱了*
  Zhāng Wěi gōujié de Lǐ Qiáng qùnián rùyùle
  Zhang Wei conspired REL Li Qiang last.year jailed
  ‘LiQiang who ZhangWei conspired with went to jail last year.’
5 *杨敏辅导*的*杨昊*今天*就任了*
  Yáng Mǐn fǔdǎo de Yáng Hào jīntiān jiùrènle
  Yang Min mentor REL Yang Hao today inducted
  ‘Yang Hao who Yang Min mentored was inducted today.’
6 *王强关注*的*王洁*上周*获胜了*
  Wáng Qiáng guānzhù de Wáng Jié shàngzhōu huòshèngle
  Wang Qiang follow.with.interest REL Wang Jie last.week won
  ‘Wang Jie who Wang Qiang follows with interest won last week.’
7 *王霞拥护*的*马超*今天*下台了*
  Wáng Xiá yōnghù de Mǎ Chāo jīntiān xiàtáile
  Wang Xia supports REL Ma Chao today resigned
  ‘Ma Chao who Wang Xia supports resigned today.’
8 *李丽抢救*的*罗英*前天*牺牲了*
  Lǐ Lì qiǎngjiù de Luó Yīng qiántiān xīshēngle
  Li Li saved REL Luo Ying day.before.yesterday sacrificed
  ‘Luo Ying who Li Li saved sacrificed his life the day before yesterday.’
9 *李昊寻找*的*王刚*已经*逝世了*
  Lǐ Hào xúnzhǎo de Wáng Gāng yǐjīng shìshìle
  Li Hao search REL Wang Gang already passed.away
  ‘Wang Gang who Li Hao is searching for has passed away already.’
10 *王军关心*的*李婧*去年*结婚了*
  Wáng Jūn guānxīn de Lǐ Jìng qùnián jiéhūnle
  Wang Jun cares.about REL Li Jing last year married
  ‘Li Jing who Wang Jun cares about married last year.’
11 *王芳推荐*的*张强*刚才*上台了*
  Wáng Fāng tuījiàn de Zhāng Qiáng gāngcái shàngtáile
  Wang Fang recommended REL Zhang Qiang just.now appear.on.stage
  ‘Zhang Qiang who Wang Fang recommended appeared on the stage just now.’
12 *李明聘请*的*刘梦*去年*转行了*
  Lǐ Míng pìnqǐng de Liú Mèng qùnián zhuǎnhángle
  Li Ming hired REL Liu Meng last.year switched.profession
  ‘Liu Meng who Li Ming hired switched to another profession last year.’
13 *王茜提及*的*李明*去年*搬家了*
  Wáng Qiàn tíjí de Lǐ Míng qùnián bānjiāle
  Wang Qian mentioned REL Li Ming last.year moved
  ‘Li Ming who Wang Qian mentioned moved last year.’
14 *李艳采访*的*王丽*昨天*自杀了*
  Lǐ Yàn cǎifǎng de Wáng Lì zuótiān zìshāle
  Li Yan interviewed REL Wang Li yesterday committed.suicide
  ‘Wang Li who Li Yan interviewed committed suicide yesterday.’
15 *李明资助*的*刘洋*去年*破产了*
  Lǐ Míng zīzhù de Liú Yáng qùnián pòchǎnle
  Li Ming funded REL Liu Yang last.year bankrupted
  ‘Liu Yang who Li Ming funded went bankrupt last year.’
16 *周婕提拔*的*张梦*上周*违纪了*
  Zhōu Jié tíbá de Zhāng Mèng shàngzhōu wéijìle
  Zhou Jie promoted REL Zhang Meng last.week broke.rules
  ‘Zhang Meng who Zhou Jie promoted broke the rules last week.’
17 *张杰负责*的*刘鹏*去年*辞职了*
  Zhāng Jié fùzé de Liú Péng qùnián cízhíle
  Zhang Jie in.charge.of REL Liu Peng last.year resigned
  ‘Liu Peng who Zhang Jie was in charge of resigned last year.’
18 *王静录用*的*李莲*今天*加班了*
  Wáng Jìng lùyòng de Lǐ Lián jīntiān jiābānle
  Wang Jing employed REL Li Lian today worked.overtime
  ‘Li Lian who Wang Jing employed worked overtime today.’
19 *王静信任*的*李涛*今天*缺席了*
  Wáng Jìng xìnrèn de Lǐ Tāo jīntiān quēxíle
  Wang Jing trust REL Li Tao today absent
  ‘Li Tao who Wang Jing trusts is absent today.’
20 *王阳培养*的*周洁*去年*掌权了*
  Wáng Yáng péiyǎng de Zhōu Jié qùnián zhǎngquánle
  Wang Yang mentored REL Zhou Jie last.year in.power
  ‘Zhou Jie who Wang Yang mentored came into power last year.’
21 *刘刚思念*的*高军*昨天*生病了*
  Liú Gāng sīniàn de Gāo Jūn zuótiān shēngbìngle
  Liu Gang missed REL Gao Jun yesterday sick
  ‘Gao Jun who Liu Gang missed was sick yesterday.’
22 *张涛招待*的*李艳*去年*退休了*
  Zhāng Tāo zhāodài de Li Yàn qùnián tuìxiūle
  Zhang Tao entertained REL Li Yan last.year retired
  ‘Li Yan who Zhang Tao entertained retired last year.’
23 *周超指导*的*李勇*刚才*出事了*
  Zhōu Chāo zhǐdǎo de Lǐ Yǒng gāngcái chūshìle
  Zhou Chao tutored REL Li Yong just.now accident
  ‘Li Yong who Zhou Chao tutored had an accident just now.’
  ‘Something bad happened to Li Yong who Zhou Chao tutored just now.’
24 *王芳栽培*的*张静*上周*去世了*
  Wáng Fāng zāipéi de Zhāng Jìng shàngzhōu qùshìle
  Wang Fang mentored REL Zhang Jing last.week died.
  ‘Zhang Jing who Wang Fang mentored died last week.’
25 *王娟支持*的*李勇*昨天*获奖了*
  Wáng Juān zhīchí de Lǐ Yǒng zuótiān huòjiǎngle
  Wang Juan support REL Li Yong yesterday won.award
  ‘Li Yong who Wang Juan supports won an award yesterday.’
26 *张超宴请*的*王婕*刚才*道歉了*
  Zhāng Chāo yànqǐng de Wáng Jié gāngcái dàoqiànle
  Zhang Chao entertained REL Wang Jie just.now apologized
  ‘Wang Jie who Zhang Chao entertained apologized just now.’
27 *张静逮捕*的*李鹏*那天*受伤了*
  Zhāng Jìng dàibǔ de Lǐ Péng nàtiān shòushāngle
  Zhang Jing arrested REL Li Peng that.day injured
  ‘Li Peng who Zhang Jing arrested was injured that day.’
28 *刘杰赏识*的*高燕*最终*受骗了*
  Liú Jié shǎngshí de Gāo Yàn zuìzhōng shòupiànle
  Liu Jie admire REL Gao Yan eventually deceived.
  ‘Gao Yan who Liu Jie admires was deceived eventually.’
29 *王强培育*的*张勇*今年*创业了*
  Wáng Qiáng péiyù de Zhāng Yǒng jīnnián chuàngyèle
  Wang Qiang mentored REL Zhang Yong this.year started.business
  ‘Zhang Yong who Wang Qiang mentored started a business this year.’
30 *王静照顾*的*李娜*上周*捐款了*
  Wáng Jìng zhàogù de Lǐ Nà shàngzhōu juānkuǎnle
  Wang Jing take.care REL Li Na last.week made.donation
  ‘Li Na who Wang Jing takes care of made a donation last week.’
31 *王猛服务*的*李刚*今年*入选了*
  Wáng Měng fúwù de Lǐ Gāng jīnnián rùxuǎnle
  Wang Meng served REL LI Gang this.year selected
  ‘Li Gang who Wang Meng served was selected this year.’
32 *李茜担心*的*陈倩*今天*住院了* 
  Lǐ Qiàn dānxīn de Chén Qiàn jīntiān zhùyuànle
  Li Qian worried.about REL Chen Qian today hospitalized
  ‘Chen Qian who Li Qian worried about is in the hospital today.’

The following sentences are all the ORC experimental items from Experiment 2. The SRC sentence condition is given only for the first item. For the unambiguous condition, the determiner + classifier region which is the first region for all of the sentences was removed. The interest regions are designated between asterisk marks (Det+Cl, ADV, N1,V1, Freq, DE, N2, V2, N3, and the remainder of the sentence).

1. ORC: *那个*昨晚*服务生揍了*一顿*的*顾客*听说过*老板*并且记得他。*

nàgè zuówǎn fúwùshēng zòule yī dùn de gùkè tīngshuōguò lǎobǎn bìngqiě jìde tā

that.one last.night waiter beat more.than.once REL customer have heard of boss and remember him

‘The customer who the waiter beat up last night has heard of the boss and remembers him.’

1. SRC: *那个*昨晚*揍了服务生*一顿*的*顾客*听说过*老板*并且记得他。*

nàgè zuówǎn zòule fúwùshēng yī dùn de gùkè tīngshuōguò lǎobǎn bìngqiě jìde tā

that.one last.night beat waiter more.than.once REL customer have.heard.of boss and remember him

‘The customer who beat up the waiter last night has heard of the boss and remembers him.’

2. *那辆*下午*摩托车追了*很久*的*轿车*发现了*记者*所以停了下来。*

nàliàng xiàwǔ mótuōchē zhuīle hěnjiǔ de jiàochē fāxiànle jìzhě suǒyǐ tíngle xiàlái

that.car afternoon motorcycle chased long.time REL car found reporter so stopped

‘The car that the motorcycle chased for a long time in the afternoon found the reporter so it stopped.’

3. *那个*今天*男孩打了*几次*的*女孩*看到了*校长*所以假装读书。*

nàgè jīntiān nánhái dǎle jǐcì de nǚhái kàndàole xiàozhǎng suǒyǐ jiǎzhuāng dúshū

that.one today boy hit several.times REL girl saw principal so pretended to read

‘The girl who the boy hit several times today saw the principal and thus pretended to read.’

4. *那辆*当时*自行车撞了*两次*的*吉普车*拦住了*警察*并且要求调查清楚。*

nàliàng dāngshí zìxíngchē zhuàngle liǎngcì de jípǔchē lánzhùle jǐngchá bìngqiě yāoqiú diàochá qīngchu

that.car then bike hit twice REL jeep stopped police and asked investigation clear

‘The jeep that the bike hit twice then stopped the police and asked for a clear investigation.’

5. *那个*刚才*男孩推了*一下*的*妇女*偷了*店员*并且打伤了她。*

nàgè gāngcái nánhái tuīle yīxià de fùnǚ tōule diànyuán bìngqiě dǎshāngle tā

that.one just.now boy pushed a.bit REL woman stole clerk and wounded her

‘The woman who the boy pushed just now stole from the clerk and wounded her.’

6. *那个*上个月*男孩邀请了*几次*的*女孩*认识*王老师*因为上过她的课。*

nàgè shànggèyuè nánhái yāoqǐngle jǐcì de nǚhái rènshi wánglǎoshī yīnwèi shàngguò tā de kè

that.one last.month boy invited several.times REL girl knows teacher.WANG because went to her class

‘That girl who the boy invited several times last month knows teacher Wong because she went to her class.’

7. *那条*去年*主人救了*好几次*的*狗*喜欢*小男孩*所以很兴奋。*

nàtiáo qùnián zhǔrén jiùle hǎojǐcì de gǒu xǐhuan xiǎonánhái suǒyǐ hěn xīngfèn

that.animal last.year master saved several.times REL dog like little.boy so it be very excited

‘The dog that the master saved several times last year likes the little boy so it was very excited.’

8. *那个*刚才*职业选手推了*一下*的*业余选手*骂了*裁判*而且威胁了他。*

nàgè gāngcái zhíyè xuǎnshǒu tuīle yīxià de yèyú xuǎnshǒu màle cáipàn érqiě wēixiéle tā

that.one just.now professional.player pushed a.bit REL amateur scolded referee and threatened him

‘The amateur who the professional player pushed a bit just now scolded the referee and threatened him.’

9. *这个*上个月*杀手监视了*一段时间*的*侦探*讨厌*当地人*所以没有寻求帮助。*

zhège shànggèyuè shāshǒu jiānshìle yīduàn shíjiān de zhēntàn tǎoyàn dāngdìrén suǒyǐ méiyǒu xúnqiú bāngzhù

this.one last.month killer watched a.while REL detective hated local so did.not ask for help

‘The detective who the killer watched for a while last month hated the locals so he did not ask for help.’

10. *那位*最近*房东抱怨了*好多次*的*住户*找了*律师*而且打算*起诉。*

nàwèi zuìjìn fángdōng bàoyuànle hǎoduōcì de zhùhù zhǎole lǜshī érqiě dǎsuàn qǐsù

that.person recently landlord complained many.times REL tenant found lawyer and intended sue

‘The tenant who the landlord complained about many times recently found a lawyer and intends to sue.’

11. *那个*上个月*教练骂了*一顿*的*球员*爱上了*女歌星*还送她礼物。*

nàgè shànggèyuè jiàoliàn màle yīdùn de qiúyuán àishàngle nǚ gēxīng hái sòng tā lǐwù

that.one last.month coach scolded more.than.once REL player fell.love.with female singer and sent her gift

‘The player who the coach scolded last month fell in love with a female singer and sent her a gift.’

12. *那位*以前*指挥家崇拜了*很久*的*作曲家*结识了*小提琴手*并且两人常见面。*

nàwèi yǐqián zhǐhuījiā chóngbàile hěnjiǔ de zuòqǔjiā jiéshìle xiǎotíqínshǒu bìngqiě liǎng rén cháng jiànmiàn

that.person before conductor respected long.time REL composer met violinist and both meet often

‘The composer who the conductor respected for a long time in the past met a violinist and they meet often.’

13. *这个*去年*电视台批评了*几次*的*女演员*很欣赏*金城武*因为他个性坦率。*

zhège qùnián diànshìtái pīpíngle jǐcì de nǚ yǎnyuán hěn xīnshǎng jīnchéngwǔ yīnwèi tā gèxìng tǎnshuài

this.one last.year TV.station criticized several.times REL actress very.appreciate Jincheng Wu because his frank personality

‘This actress who the TV station criticized several times last year appreciates Jincheng Wu very much because of his frank personality.’

14. *那位*上个月*飞行员约了*两次*的*空姐*惹怒了*经理*因为她常迟到。*

nàwèi shànggèyuè fēixíngyuán yuēle liǎngcì de kōngjiě rěnùle jīnglǐ yīnwèi tā cháng chídào

that.person last.month pilot ask out twice REL stewardess angered manager because she often late

‘The stewardess who the pilot asked out twice last month angered the manger because she was often late.’

15. *这位*今天*导演称赞了*多次*的*男明星*批评了*影评家*并且表示很难过。*

zhèwèi jīntiān dǎoyǎn chēngzànle duōcì de nán míngxīng pīpíngle yǐngpíngjiā bìngqiě biǎoshì hěn nánguò

this.one today director praised many.times REL male star criticized critics and said he was very sad

‘The male star who the director praised many times today criticized the critics and said he was very sad.’

16. *那位*昨天*作家采访了*两个小时*的*记者*质疑了*县长候选人*而且扬言报复。*

nàwèi zuótiān zuòjiā cǎifǎngle liǎng gè xiǎoshí de jìzhě zhíyíle xiàn zhǎng hòuxuǎnrén érqiě yángyán bàofù

that.person yesterday writer interviewed two.hours REL reporter questioned county.magistrate candidate and threatened revenge

‘The reporter who the writer interviewed for two hours yesterday questioned the county magistrate candidate and threatened revenge’

17. *那个*今早*犯人追了*一阵*的*小狗*嗅出*主人*并且停了下来。*

nàgè jīnzǎo fànrén zhuīle yīzhèn de xiǎo gǒu xiùchū zhǔrén bìngqiě tíngle xiàlái

that.one this.morning criminal chased a.while REL puppy sniffed.recognize the master and stopped

‘The puppy who the prisoner chased a while this morning sniffed and recognized the mater and stopped.’

18. *那位*昨天*邻居教训了*一番*的*大妈*通知*管理员*然后诉了苦。*

nàwèi zuótiān línjū jiàoxunle yī fān de dàmā tōngzhī guǎnlǐ yuán ránhòu sule kǔ

that.person yesterday neighbor taught for.a.while REL aunt noticed administrator and complained

‘The aunt who the neighbor taught a lesson yesterday noticed the administrator and complained.’

19. *这位*去年*外交部访问了*一次*的*政治家*支持*外交官*并且相信他。*

zhèwèi qùnián wàijiāobù fǎngwènle yīcì de zhèngzhì jiā zhīchí wàijiāoguān bìngqiě xiāngxìn tā

this.person last.year ministry.foreign.affairs visited once REL politician support diplomat and believed him.

‘This politician who the Ministry of Foreign Affairs visited once last year supports the diplomat and believes him.’

20. *那个*今年*作家批评了*一番*的*评论家*洽询了*出版商*而且建议了出版内容。*

nàgè jīnnián zuòjiā pīpíngle yīfān de pínglùnjiā qiàxúnle chūbǎnshāng érqiě jiànyìle chūbǎn nèiróng

that.one this.year writer criticized for.a.while REL critic consulted publisher and suggested publication.content

‘The critic who the writer criticized for a while this year consulted the publisher and suggested the content for publication.’

21. *那位*下午*学生称赞了*多次*的*老师*认出*家长*然后打招呼。*

nàwèi xiàwǔ xuéshēng chēngzànle duōcì de lǎoshī rènchū jiāzhǎng ránhòu dǎzhāohū

that.person afternoon student praised several.times REL teacher recognized parents and.then greeted

‘The teacher who the student praised several times this afternoon recognized the parents and greeted them.’

22. *那位*上周*护士请教了*一次*的*营养师*看了*医生*而且确定病征。*

nàwèi shàngzhōu hùshì qǐngjiàole yīcì de yíngyǎngshī kànle yīshēng érqiě quèdìng bìngzhēng

that.person last.week nurse consulted once REL nutritionist went.to.see doctor and identified symptoms

‘That nutritionist who the nurse consulted once last week went to see a doctor and identified symptoms.’

23.*这位*上个月*新同事介绍了*几次*的*员工*说服过*上司*然后转了部门。*

zhèwèi shànggèyuè xīn tóngshì jièshàole jǐcì de yuángōng shuōfúguò shàngsi ránhòu zhuǎnle bùmén

this.person last.month new.colleague introduced several.times REL employee persuaded boss and.then transferred department

‘This employee who the new colleague introduced several times last month persuaded the boss and then transferred to another department.’

24. *这位*上午*妻子陪了*许久*的*丈夫*问了*旅行社*然后决定规划旅行。*

zhèwèi shàngwǔ qīzi péile xǔjiǔ de zhàngfū wènle lǚxíngshè ránhòu juédìng guīhuà lǚxíng

this.person morning wife accompany long.time REL husband inquire travel.agency and.then decide plan trip

‘The husband who his wife accompanied a long time this morning inquired the travel agency and then decided to plan a trip.’

25. *那个*上午*店员询问了*一番*的*女孩*找到*妈妈*然后回了家。*

nàgè shàngwǔ diànyuán xúnwènle yī fān de nǚhái zhǎodào māmā ránhòu huíle jiā

that.one morning clerk asked for.a.while REL girl found mom and.then returned home

‘The girl who the clerk asked about in the morning found the mom and then returned home.’

26. *这个*去年*新娘恭喜了*几次*的*商人*吻了*女儿*并且举办喜宴。*

zhège qùnián xīnniáng gōngxǐle jǐcì de shāngrén wěnle nǚ'ér bìngqiě jǔbàn xǐyàn

this.one last.year bride congratulated several.times REL businessman kissed daughter and held wedding banquet

‘This businessman who the bride congratulated several times last year kissed the daughter and held a wedding banquet.’

27. *这位*昨晚*科学家相信了*一时*的*播报员*联系*物理专家*并且调查了真相。*

zhèwèi zuówǎn kēxuéjiā xiāngxìnle yīshí de bōbàoyuán liánxì wùlǐ zhuānjiā bìngqiě diàochále zhēnxiàng

this.person last.night scientist believed momentarily REL announcer contacted physician and investigated truth

‘This announcer who the scientist believed momentarily last night contacted a physician and investigated the truth.’

28. *那个*前天*小说家取悦了*许久*的*歌剧家*想到*大学教授*所以联络了。*

nàgè qiántiān xiǎoshuōjiā qǔyuèle xǔjiǔ de gējùjiā xiǎngdào dàxué jiàoshòu suǒyǐ liánluòle

that.one day.before.yesterday novelist flatter long.time REL opera.singer thought.of university.professor so contacted

‘The opera singer who the novelist flattered for a long time the day before yesterday thought of the university professor so she contacted him.’

29. *这个*刚才*姑姑接了*一次*的*叔叔*送了*伯伯*并且一起坐车去。*

zhège gāngcái gūgū jiēle yīcì de shūshu sòngle bóbo bìngqiě yīqǐ zuòchē qù

this.one just.now aunt picked up once REL the.younger.uncle sent the.older.uncle and went by car

‘The younger uncle who the aunt picked up once just now sent off the older uncle and went by car together.’

30. *这个*今晚*客人迎接了*多次*的*男生*遇见*一位女孩*然后要了电话号码。*

zhège jīnwǎn kèrén yíngjiēle duōcì de nánshēng yùjiàn yīwèi nǚhái ránhòu yàole diànhuà hàomǎ

this.one tonight guest greeted many.times REL boy met one.girl and.then asked for phone.number

‘This boy who the guest greeted many times tonight met a girl and then asked for her phone number.’

31. *那个*刚刚*女同学邀请了*一次*的*男生*拒绝*好朋友*并且离开了教室。*

nàgè gānggāng nǚ tóngxué yāoqǐngle yīcì de nánshēng jùjué hǎopéngyǒu bìngqiě líkāile jiàoshì

that.one just.now female.classmate invited once REL boy refused good.friend and left classroom

‘The boy who the female classmate invited once just now refused a good friend and left the classroom.’

32. *那位*昨天*亲戚拜访了*两次*的*生意人*撞到*青梅竹马*并且感到讶异。*

nàwèi zuótiān qīnqi bàifǎngle liǎngcì de shēngyìrén zhuàngdào qīngméizhúmǎ bìngqiě gǎndào yà yì

that.person yesterday relatives visited twice REL businessman hit childhood friend and felt surprised

‘The businessman who the relatives visited twice yesterday hit the childhood friend and felt surprised.’

## Supporting information

S1 Tables These are the tables for Experiment 1 and 2. Within S1 Tables, Tables A-to-H are provided. Tables A to C detail the means and LME models for Experiment 1. The remainder of the tables detail the means and LME models for Experiment 2.
(PDF)

S1 Data This is the eye-tracking data for Experiments 1 and 2. (XLSX)
