# Correction: Ambiguity in the processing of Mandarin Chinese relative clauses: One factor cannot explain it all

**DOI:** 10.1371/journal.pone.0186618

**Published:** 2017-10-12

**Authors:** Michael P. Mansbridge, Katsuo Tamaoka, Kexin Xiong, Rinus G. Verdonschot

There is an error in the supplementary file S1 Tables. Within Table A, the variable names under RC condition are labeled incorrectly as “Plausibility” and “Verification”. They should instead read “ORC” and “SRC” respectively. Please view the correct S1 Tables below.

## Supporting information

S1 Tables These are the tables for Experiment 1 and 2. Within S1 Tables, Tables A-to-H are provided. Tables A to C detail the means and LME models for Experiment 1. The remainder of the tables detail the means and LME models for Experiment 2.
(PDF)
