# Supplementary material for: Ambiguity in the processing of Mandarin Chinese relative clauses: One factor cannot explain it all
Source: PLoS One. 2017 Jun 8;12(6):e0178369. doi: 10.1371/journal.pone.0178369 (PMC5464565; doi:10.1371/journal.pone.0178369)
Supplement: S1 Tables — Within S1 Tables, Tables A-to-H are provided. Tables A to C detail the means and LME models for Experiment 1. The remainder of the tables detail the means and LME models for Experiment 2. (PDF) [file pone.0178369.s001.pdf]

Table A. Experiment 1: RC condition and Task type means

	RC Condition				Task Type			
	Plausibility		Verification		Plausibility		Verification	
	Mean	SE	Mean	SE	Mean	SE	Mean	SE
Sentence								
TT	2,819	40	2,860	42	2,372	31	3,324	43
ACC	92.01 %	0.92%	89.58%	1.04%	92.13%	0.92%	89.47%	1.04%
N1V1(RC)								
FP	744	11	731	11	665	10	814	12
RR	805	23	820	25	556	16	1002	24
RI	76.57%	1.50%	78.55%	1.48%	64.82%	1.69%	90.67%	1.05%
DE (Relativizer)								
FP	206	3	226	4	213	3	220	4
RR	292	14	305	13	287	16	306	12
GP	286	10	334	10	270	8	348	12
RO	11.60%	1.54%	18.70%	1.76%	11.90%	1.55%	18.52%	1.76%
RI	36.19%	2.32%	36.59%	2.17%	27.46%	2.14%	44.44%	2.26%
N2 (Head Noun)								
FP	322	5	327	6	289	4	362	6
RR	515	16	512	18	364	13	593	16
GP	446	14	504	17	325	7	630	20
RO	15.36%	1.29%	20.48%	1.47%	7.50%	0.95%	28.37%	1.63%
RI	28.30%	1.61%	24.04%	1.55%	15.27%	1.29%	37.25%	1.75%
ADV (Adverb)								
FP	311	6	311	5	322	6	299	5
RR	377	14	369	14	367	13	380	15
GP	646	23	703	28	554	18	813	31
RO	36.38%	1.78%	37.29%	1.83%	29.23%	1.65%	45.45%	1.92%
RI	12.53%	1.22%	14.71%	1.34%	13.24%	1.23%	14.01%	1.34%
V2 (Matrix Verb)								
FP	352	10	363	11	379	11	330	9
RR	385	28	361	25	404	26	341	26
RO	80.37%	1.71%	81.84%	1.72%	71.31%	1.87%	93.81%	1.14%
N1 V1 DE (Full RC)								
FP	829	12	874	12	770	10	936	12
RR	852	23	885	27	594	16	1,083	26
RI	79.97%	1.42%	80.23%	1.43%	69.22%	1.64%	91.32%	1.01%
N2 ADV V2 (Matrix Clause)								
FP	915	16	874	17	910	16	879	17
RR	812	27	832	28	721	25	889	28
RO	79.97%	1.42%	80.44%	1.43%	69.22%	1.64%	91.56%	1.00%

Note. Means and standard errors of reading times are displayed in milliseconds.

Table B. Experiment 1: RC condition : Task type interaction means

	Plausibility				Verification			
	ORC		SRC		ORC		SRC	
	Mean	SE	Mean	SE	Mean	SE	Mean	SE
Sentence								
TT	2,320	44	2,422	44	3,309	57	3,341	64
ACC	91.20%	1.36%	93.06%	1.22%	92.82%	1.24%	86.11%	1.67%
N1V1 (RC)								
FP	653	13	678	15	836	17	789	16
RR	566	24	547	21	967	32	1,041	37
RI	62.18%	2.45%	67.41%	2.34%	90.75%	1.45%	90.59%	1.52%
DE (Relativizer)								
FP	205	4	220	5	208	5	232	5
RR	278	25	295	21	300	17	310	17
GP	249	10	288	11	318	16	375	16
RO	8.00%	1.92%	15.19%	2.34%	14.72%	2.34%	21.96%	2.60%
RI	25.50%	3.09%	29.11%	2.96%	45.45%	3.28%	43.53%	3.11%
N2 (Head Noun)								
FP	288	6	290	6	356	8	368	10
RR	388	20	342	16	580	21	608	24
GP	312	8	337	10	579	25	684	31
RO	4.96%	1.11%	10.00%	1.52%	25.38%	2.18%	31.61%	2.43%
RI	18.80%	2.00%	11.79%	1.64%	37.44%	2.43%	37.06%	2.52%
ADV (Adverb)								
FP	320	8	323	8	301	8	296	7
RR	375	20	359	16	378	20	381	23
GP	567	27	540	24	733	37	902	52
RO	29.92%	2.35%	28.53%	2.31%	43.34%	2.64%	47.80%	2.81%
RI	11.29%	1.62%	15.18%	1.84%	13.88%	1.84%	14.15%	1.96%
V2 (Matrix Verb)								
FP	368	15	390	15	335	12	324	14
RR	454	41	355	31	321	35	370	40
RO	69.44%	2.72%	73.09%	2.56%	92.86%	1.63%	95.00%	1.54%
N1 V1 DE (Full RC)								
FP	733	14	806	16	923	18	949	17
RR	603	24	585	22	1,031	33	1,141	41
RI	67.26%	2.37%	71.14%	2.26%	92.50%	1.32%	90.05%	1.55%
N2 ADV V2 (Matrix Clause)								
FP	926	22	894	22	903	24	853	25
RR	700	37	740	34	880	37	898	41
RO	67.26%	2.37%	71.14%	2.26%	92.50%	1.32%	90.54%	1.52%

Note. Means and standard errors of reading times are displayed in milliseconds.

Table C. Experiment 1: linear mixed effect models

	RC Condition			Task Type			Interaction		
	coef.	SE	*t/z* value	coef.	SE	*t/z* value	coef.	SE	*t/z* value
Sentence									
TT	0.03	0.01	1.82 †	0.34	0.01	23.47***	-0.04	0.03	-1.23
ACC	-0.26	0.17	-1.51	-0.29	0.17	-1.70 †	-1.05	0.35	-3.05**
N1 V1 (RC)									
FP	-0.01	0.02	-0.86	0.20	0.02	12.26***	-0.09	0.03	-2.67**
RR	0.01	0.03	0.39	0.60	0.03	17.90***	0.13	0.07	1.95 †
RI	0.13	0.15	0.89	1.78	0.15	11.85***	-0.26	0.30	-0.89
DE (Relativizer)									
FP	0.09	0.02	4.20***	0.03	0.02	1.39	0.04	0.04	0.96
RR	0.04	0.06	0.61	0.05	0.06	0.87	-0.03	0.12	-0.23
GP	0.15	0.04	4.41***	0.19	0.04	5.38***	0.06	0.07	0.87
RO	0.61	0.20	3.03**	0.61	0.20	3.01**	-0.18	0.40	-0.44
RI	0.06	0.14	0.40	0.80	0.15	5.49***	-0.30	0.29	-1.05
N2 (Head Noun)									
FP	0.01	0.02	0.51	0.19	0.02	9.75***	0.02	0.04	0.42
RR	-0.02	0.04	-0.58	0.47	0.04	11.61***	0.15	0.08	1.84 †
GP	0.09	0.03	3.23**	0.49	0.03	17.55***	0.09	0.06	1.65 †
RO	0.59	0.17	3.40***	1.89	0.18	10.59***	-0.44	0.34	-1.27
RI	-0.30	0.13	-2.34*	1.29	0.13	9.77***	0.56	0.26	2.16*
ADV (Adverb)									
FP	0.002	0.02	0.10	-0.07	0.02	-3.32***	-0.02	0.04	-0.42
RR	-0.03	0.05	-0.59	-0.01	0.05	-0.10	0.01	0.10	0.07
GP	0.05	0.04	1.16	0.27	0.04	6.59***	0.15	0.08	1.85 †
RO	0.05	0.12	0.38	0.82	0.12	6.77***	0.24	0.24	1.00
RI	0.21	0.16	1.33	0.06	0.16	0.39	-0.30	0.32	-0.94
V2 (Matrix Verb)									
FP	0.03	0.03	0.76	-0.10	0.03	-2.89**	-0.12	0.07	-1.73 †
RR	-0.04	0.09	-0.42	-0.20	0.09	-2.13*	-0.05	0.07	-0.70
RO	0.28	0.23	1.26	1.92	0.23	8.42***	0.22	0.45	0.49
N1 V1 DE (Full RC)									
FP	0.06	0.01	4.29***	0.19	0.01	13.03***	-0.05	0.03	-1.73
RR	0.03	0.03	0.93	0.61	0.03	18.90***	0.15	0.06	2.29*
RI	-0.05	0.15	-0.31	1.65	0.15	10.67***	-0.51	0.31	-1.67
N2 ADV V2 (Matrix Clause)									
FP	-0.06	0.02	-2.64**	-0.10	0.02	-3.91***	-0.03	0.05	-0.56
RR	0.06	0.04	1.35	0.18	0.05	4.07***	-0.06	0.09	-0.71
RO	-0.02	0.15	-0.13	1.68	0.16	10.75***	-0.45	0.31	-1.47

Note. p < .1 † , p < .05*, p < .01**, p < .001***. A positive coefficient indicates an increase in reading time, accuracy or regression for the SRC condition or Verification task.

Table D. Experiment 2: RC condition and Determiner type means

	RC Condition				Determiner Type			
	ORC		SRC		Empty		DCL	
	Mean	SE	Mean	SE	Mean	SE	Mean	SE
Sentence								
TT	5,571	111	4,993	103	5,152	107	5,413	109
ACC	79.3%	1.7%	77.9%	1.7%	78.5%	1.7%	78.8%	1.7%
N1V1(RC)								
FP	510	16	545	17	548	16	508	17
RR	1,040	36	873	32	985	34	931	35
GP	869	27	818	24	783	21	905	29
RO	34.8%	2.2%	26.2%	2.1%	25.8%	2.0%	35.3%	2.2%
RI	75.8%	2.0%	73.8%	2.1%	80.8%	1.8%	68.8%	2.2%
Freq (Frequency Phrase)								
FP	264	7	267	6	277	7	253	6
RR	402	16	377	17	414	16	361	16
GP	338	11	330	11	350	11	318	10
RO	10.3%	1.5%	11.8%	1.6%	10.4%	1.5%	11.7%	1.6%
RI	49.2%	2.4%	45.1%	2.4%	51.3%	2.4%	42.8%	2.4%
DE (Relativizer)								
FP	238	5	218	5	232	5	225	5
RR	264	12	282	17	294	14	242	12
GP	360	18	308	14	351	17	318	16
RO	17.2%	2.3%	13.4%	2.1%	15.0%	2.2%	15.7%	2.3%
RI	36.9%	3.0%	25.3%	2.7%	32.6%	2.9%	29.9%	2.9%
N2 (Head Noun)								
FP	287	7	290	7	289	7	288	7
RR	488	20	421	18	477	20	433	19
GP	409	16	403	16	420	17	391	15
RO	20.1%	1.9%	14.7%	1.7%	18.4%	1.8%	16.6%	1.8%
RI	40.7%	2.3%	34.3%	2.3%	37.9%	2.3%	37.3%	2.3%
V2 (Matrix Verb)								
FP	335	9	331	9	338	9	328	8
RR	506	20	433	19	490	22	453	18
GP	459	18	422	16	469	19	414	16
RO	16.1%	1.7%	14.2%	1.7%	17.4%	1.8%	13.0%	1.6%
RI	42.6%	2.3%	45.1%	2.4%	42.5%	2.4%	45.2%	2.4%
N3 (Matrix Object)								
FP	312	8	299	8	301	8	310	8
RR	427	19	425	21	437	22	415	18
GP	435	15	442	16	436	16	441	15
RO	21.0%	2.0%	23.5%	2.1%	23.7%	2.1%	20.8%	2.0%
RI	29.8%	2.2%	29.4%	2.2%	28.6%	2.2%	30.7%	2.2%

Note. Means and standard errors of reading times are displayed in milliseconds.

Table E. Experiment 2: RC condition : Determiner type interaction means

	Empty				DCL			
	ORC		SRC		ORC		SRC	
	Mean	SE	Mean	SE	Mean	SE	Mean	SE
Sentence								
TT	5,424	155	4,872	143	5,719	159	5,111	149
ACC	79.8%	2.4%	77.1%	2.5%	78.8%	2.4%	78.8%	2.4%
N1V1 (RC)								
FP	529	21	566	24	491	24	525	24
RR	1,061	50	907	44	1,019	53	836	46
GP	735	26	832	33	1,006	46	804	36
RO	25.0%	2.8%	26.5%	2.9%	44.8%	3.3%	25.8%	2.9%
RI	83.6%	2.4%	77.9%	2.8%	67.8%	3.1%	69.9%	3.0%
Freq (Frequency Phrase)								
FP	281	10	272	9	245	8	261	8
RR	424	21	401	26	371	24	352	22
GP	365	17	334	15	310	14	326	15
RO	10.4%	2.1%	10.5%	2.1%	10.2%	2.1%	13.2%	2.4%
RI	57.9%	3.3%	44.3%	3.4%	39.8%	3.4%	45.9%	3.5%
DE (Relativizer)								
FP	242	7	221	7	233	7	216	6
RR	281	17	316	26	243	15	240	20
GP	380	27	320	19	339	25	296	20
RO	16.1%	3.1%	13.8%	3.0%	18.3%	3.4%	13.0%	3.0%
RI	42.3%	4.2%	22.3%	3.7%	31.3%	4.1%	28.5%	4.1%
N2 (Head Noun)								
FP	287	10	292	10	287	10	288	10
RR	522	31	428	23	451	25	413	28
GP	431	24	409	23	386	20	397	21
RO	23.7%	2.8%	12.7%	2.3%	16.4%	2.5%	16.7%	2.6%
RI	40.8%	3.3%	34.7%	3.3%	40.6%	3.3%	34.0%	3.2%
V2 (Matrix Verb)								
FP	339	13	337	12	331	12	325	12
RR	542	31	435	30	472	26	432	25
GP	494	29	442	23	425	22	403	22
RO	18.4%	2.6%	16.2%	2.5%	13.8%	2.3%	12.3%	2.2%
RI	40.8%	3.3%	44.3%	3.4%	44.4%	3.3%	45.9%	3.4%
N3 (Matrix Object)								
FP	306	11	295	12	317	10	304	11
RR	438	30	435	33	416	25	414	27
GP	435	21	437	24	436	21	446	23
RO	20.7%	2.8%	26.7%	3.1%	21.2%	2.8%	20.3%	2.8%
RI	28.6%	3.1%	28.6%	3.1%	31.1%	3.2%	30.2%	3.2%

Note. Means and standard errors of reading times are displayed in milliseconds.

Table F. Experiment 2: linear mixed effect models

	RC Condition			Determiner Type			Interaction		
	coef.	SE	*t/z* value	coef.	SE	*t/z* value	coef.	SE	*t/z* value
Sentence									
TT	-0.11	0.02	-6.23***	0.02	0.01	3.13**	0	0.01	0.37
ACC	-0.1	0.15	-0.65	0.01	0.05	0.1	0.06	0.1	0.63
N1 V1 (RC)									
FP	0.07	0.04	1.83 †	-0.04	0.01	-2.90**	0.02	0.03	0.79
RR	-0.19	0.05	-4.32***	-0.03	0.02	-1.87 †	-0.02	0.03	-0.67
GP	-0.05	0.03	-1.52	0.03	0.01	2.35*	-0.1	0.02	-4.46***
RO	-0.42	0.15	-2.73**	0.15	0.05	2.97**	-0.34	0.1	-3.35***
RI	-0.18	0.17	-1.04	-0.27	0.06	-4.62***	0.17	0.11	1.47
Freq (Frequency Phrase)									
FP	0.04	0.03	1.34	-0.02	0.01	-2.41*	0.03	0.02	1.79 †
RR	-0.07	0.05	-1.46	-0.04	0.02	-2.40*	0.03	0.03	0.9
GP	-0.01	0.04	-0.2	-0.03	0.01	-2.43*	0.05	0.02	2.00*
RO	0.15	0.23	0.65	0.03	0.08	0.41	0.09	0.15	0.61
RI	-0.23	0.15	-1.51	-0.12	0.05	-2.43*	0.31	0.1	3.05**
DE (Relativizer)									
FP	-0.06	0.03	-2.36*	-0.01	0.01	-0.59	0.01	0.02	0.82
RR	0.04	0.07	0.61	-0.07	0.02	-3.10**	-0.04	0.04	-1.02
GP	-0.11	0.05	-2.27*	-0.03	0.02	-1.64	0.01	0.03	0.47
RO	-0.3	0.25	-1.2	0.02	0.08	0.26	-0.08	0.17	-0.5
RI	-0.54	0.2	-2.79**	-0.03	0.07	-0.43	0.27	0.13	2.10*
N2 (Head Noun)									
FP	0.02	0.03	0.68	0	0.01	-0.56	0	0.02	0.28
RR	-0.12	0.05	-2.51*	-0.04	0.02	-2.22*	0.01	0.03	0.25
GP	-0.01	0.04	-0.4	-0.02	0.01	-1.99*	0.02	0.02	0.96
RO	-0.39	0.19	-2.09*	-0.03	0.06	-0.49	0.27	0.12	2.21*
RI	-0.35	0.15	-2.31*	-0.01	0.05	-0.11	-0.01	0.1	-0.08
V2 (Matrix Verb)									
FP	0.00	0.03	-0.06	-0.01	0.01	-1.03	-0.01	0.02	-0.35
RR	-0.14	0.05	-2.79**	-0.01	0.02	-0.77	0.07	0.03	2.07*
GP	-0.05	0.04	-1.26	-0.04	0.01	-2.86**	0	0.03	0.04
RO	-0.16	0.19	-0.82	-0.12	0.06	-1.88 †	0.01	0.13	0.1
RI	0.09	0.15	0.57	0.04	0.05	0.88	-0.03	0.1	-0.32
N3 (Matrix Object)									
FP	-0.05	0.03	-1.90 †	0.01	0.01	1.49	0.01	0.02	0.81
RR	-0.03	0.06	-0.46	0	0.02	0	-0.01	0.04	-0.17
GP	-0.02	0.04	-0.58	0	0.01	0.1	0.02	0.03	0.88
RO	0.14	0.17	0.83	-0.06	0.06	-1.12	-0.12	0.11	-1.05
RI	-0.02	0.16	-0.11	0.03	0.05	0.62	-0.02	0.11	-0.14

Note. A positive coefficient indicates an increase in reading time for SRCs or for the DCL type. $p < .1$ †, $p < .05$*, $p < .01$**, $p < .001$***

Table G. Experiment 2: means for the additional analyses

	RC Condition				Determiner Type			
	ORC		SRC		Empty		DCL	
	Mean	SE	Mean	SE	Mean	SE	Mean	SE
N1 V1 Freq DE (Full RC)								
FP	754	20	798	20	807	17	713	25
RR	1,533	42	1,312	37	1,460	34	1,357	50
GP	1,211	27	1,174	23	1,165	20	1,251	35
RO	33.2%	1.8%	27.3%	1.7%	27.0%	1.5%	36.7%	2.3%
RI	83.3%	1.4%	79.6%	1.5%	84.5%	1.2%	75.4%	2.0%
N2 V2 N3 (Matrix Clause)								
FP	808	19	831	19	806	17	846	24
RR	1,190	36	988	33	1,142	32	994	39
GP	1,260	31	1,139	26	1,236	26	1,129	32
RO	29.3%	1.7%	22.6%	1.6%	28.5%	1.5%	20.9%	1.9%
RI	53.4%	1.9%	52.9%	1.9%	51.6%	1.7%	56.3%	2.3%

	Empty				DCL			
	ORC		SRC		ORC		SRC	
	Mean	SE	Mean	SE	Mean	SE	Mean	SE
N1 V1 Freq DE (Full RC)								
FP	795	25	820	25	673	35	754	35
RR	1,562	51	1,350	46	1,474	76	1,231	64
GP	1,138	30	1,192	27	1,363	55	1,139	42
RO	26.2%	2.0%	27.9%	2.1%	47.4%	3.3%	26.1%	2.9%
RI	88.0%	1.5%	81.0%	1.8%	73.9%	2.9%	77.0%	2.8%
N2 V2 N3 (Matrix Clause)								
FP	771	23	842	24	883	35	810	33
RR	1,254	46	1,017	42	1,053	55	934	54
GP	1,321	41	1,148	32	1,137	45	1,121	44
RO	33.9%	2.2%	23.0%	2.0%	20.0%	2.6%	21.7%	2.7%
RI	51.9%	2.3%	51.3%	2.4%	56.5%	3.3%	56.1%	3.3%

Table H. Experiment 2: linear mixed effect models for the additional analyses

	RC Condition			Determiner Type			Interaction		
	coef.	SE	*t/z* value	coef.	SE	*t/z* value	coef.	SE	*t/z* value
N1 V1 Freq DE (Full RC)									
FP	0.12	0.04	2.98**	-0.06	0.01	-4.29***	0.05	0.03	1.82 †
RR	-0.19	0.04	-4.98***	-0.03	0.01	-2.43*	0.00	0.02	0.009
GP	-0.04	0.02	-1.821 †	0.01	0.01	1.437	-0.07	0.01	-5.02***
RO	-0.51	0.13	-3.78***	0.16	0.04	3.53***	-0.39	0.09	-4.38***
RI	-0.26	0.16	-1.66 †	-0.25	0.05	-4.76***	0.28	0.10	2.74**
N2 V2 N3 (Matrix Clause)									
FP	0.01	0.03	0.468	0.01	0.01	1.238	-0.06	0.02	-2.70**
RR	-0.18	0.04	-4.46***	-0.04	0.01	-3.15**	0.03	0.03	1.102
GP	-0.08	0.03	-2.96**	-0.03	0.01	-3.41***	0.04	0.02	2.57*
RO	-0.25	0.15	-1.71 †	-0.16	0.05	-3.26**	0.25	0.10	2.59**
RI	-0.06	0.13	-0.439	0.07	0.04	1.71 †	0.01	0.08	0.073

Note. A positive coefficient indicates an increase in reading time for the SRC condition or an increase in reading time for the DCL type.
